# Long-read sequencing of *FLG* identifies extensive copy number variation in exon 3 and detects rare loss-of-function variants

**DOI:** 10.1016/j.xjidi.2026.100522

**Published:** 2026-08-06

**Authors:** Victor Grentzinger, Maria Artesi, Leonor Palmeira, Nathalie Renotte, Nadine Cambisano, Vinciane Dideberg, Laura Helou, Benoit Charloteaux, Bita Dezfoulian, Gilles Absil, Justine Pêtre, Murielle Sabatiello, Colombine Meunier, W.H. Irwin McLean, Aileen Sandilands, Maeve A. McAleer, Alan D. Irvine, Annette Uwineza, Alice Amani Uwajeni, Sabine Olivier, Keith Durkin, Vincent Bours

**Affiliations:** 1 Laboratory of Human Genetics, GIGA Research Institute, Liège, Belgium;; 2 Center of Genetics, University Hospital, Liège, Belgium; 3 Department of Pediatric Dermatology, Clinique CHC MontLegia, Liège, Belgium; 4 Center for Medical Genetics, Institut de Pathologie et de Génétique Gosselies, Charleroi, Belgium; 5 Dermatology and Genetic Medicine, Division of Biological Chemistry and Drug Discovery, School of Life Sciences, University of Dundee, Dundee, United Kingdom; 6 Clinical Medicine, Trinity College Dublin, Dublin, Ireland; 7 Department of Biochemistry, Molecular Biology and Genetics, University of Rwanda, Kigali, Rwanda; 8 Department of Dermatology, Centre Hospitalier University of Kigali (CHUK), Kigali, Rwanda; 9 Theratopix Pharma S.A., Liège, Belgium

**Keywords:** Atopic dermatitis, Bioinformatics, Genomic diversity, Skin barrier, Tandem repeats

## Abstract

Loss-of-function (LOF) variants of the *FLG* gene are risk factors for atopic dermatitis. However, owing to the repetitive nature of exon 3, these variants are difficult to characterize. We sought to determine whether the use of long-read technologies would enable better characterization of the *FLG* gene and improve the detection of LOF variants. We sequenced a diverse clinical cohort of 192 samples collected in Belgium, Ireland, and Rwanda using long-read sequencing as well as publicly available data. We identified both common and rare LOF variants linked to atopic dermatitis in our clinical cohort, a number of compound heterozygotes, as well as 1 previously undescribed LOF variant. We also observed 20 previously undescribed *FLG* alleles, which varied in length by up to 13 kb. These were mostly found in samples of sub-Saharan origin and appear to be products of nonallelic homologous recombination. Despite the use of a long-read–based approach, we did not detect any *FLG* LOF variants in our African cohort, highlighting differences in *FLG* LOF burden and atopic dermatitis genetic risk between populations. Our approach has the potential to be a useful tool to identify *FLG* variants contributing to atopic dermatitis in various human populations.

## Introduction

Atopic dermatitis (AD) is a complex and chronic inflammation/irritation of the skin that can be provoked by disruption of the skin’s most superficial layer, the stratum corneum (Angelova-Fischer et al, 2011; Brown and McLean, 2012; Osawa et al, 2011; Stefanovic and Irvine, 2024). Loss-of-function (LOF) variants throughout the *FLG* gene can induce this disruption and are thus AD genetic predisposing factors (Brown and McLean, 2012; Osawa et al, 2011; Palmer et al, 2006; Stefanovic and Irvine, 2024). *FLG* codes for the pro-FLG protein, which is essential for maintaining the integrity of the epidermis (Angelova-Fischer et al, 2011; Gan et al, 1990). Its structure is composed of successive FLG monomers, which, after release, bind to the keratin fibers to create and maintain the stratum corneum (Brown and McLean, 2012). LOF variants produce a truncated pro-FLG missing the C-terminal domain, an essential factor for the proteolysis and release of the FLG monomers (Khatib et al, 2024; Margolis et al, 2018; Palmer et al, 2006; Sandilands et al, 2007; Smith et al, 2006). LOF variants in *FLG* are semidominant, and AD severity is generally higher in homozygotes or compound heterozygotes (Lassen et al, 2024; Smith et al, 2006). Patients with AD carrying *FLG* LOF variants often show moderate-to-severe AD, with an early onset; a reduced QOL; and increased risk of skin infections and other atopic diseases, such as asthma or food allergies (Irvine and Mina-Osorio, 2019; Lassen et al, 2024; Mulick et al, 2021). The characterization of LOF variants within the *FLG* gene sequence is thus of major interest, but owing to the structure of the gene, this can be technically challenging (Mandelker et al, 2016). The FLG monomers are encoded by a repetitive region on the third *FLG* exon, which leads to high intragenic similarity (Gan et al, 1990). Moreover, multiple copy number variations (CNVs) were identified at that location, with 7 different *FLG* alleles described in the literature (Brown and McLean, 2012; Eaaswarkhanth et al, 2016; Mukamel et al, 2021; Yuda et al, 2025). Previous work described 4 different alleles containing between 10 and 12 monomers (Brown and McLean, 2012; Eaaswarkhanth et al, 2016; Mukamel et al, 2021) (Figure 1), and a recent paper described 3 previously undescribed alleles (Yuda et al, 2025). To date, approximately 170 pathogenic/likely pathogenic LOF variants have been identified in different human populations and listed in the Genome Aggregation Database (Chen et al, 2024). However, this is likely to be an underestimate, because the *FLG* gene belongs to the so-called “NGS Dead Zone” list of medically relevant genes (Mandelker et al, 2016; Wagner et al, 2022). Different approaches have been developed to reduce the cost and simplify the sequencing of this gene (Van Leersum et al, 2020; Wong et al, 2018); however, they still rely on short-read technologies and thus do not solve the limitations of short reads in repetitive regions. Furthermore, *FLG* LOF variants are known to be population specific (Margolis et al, 2019): most described variants come from European and Asian populations (Sandilands et al, 2007; Wong et al, 2018), whereas variants specific to African populations appear to be rare (Margolis et al, 2018; Stefanovic and Irvine, 2024; Thawer-Esmail et al, 2014; Winge et al, 2011).

**Figure 1:**
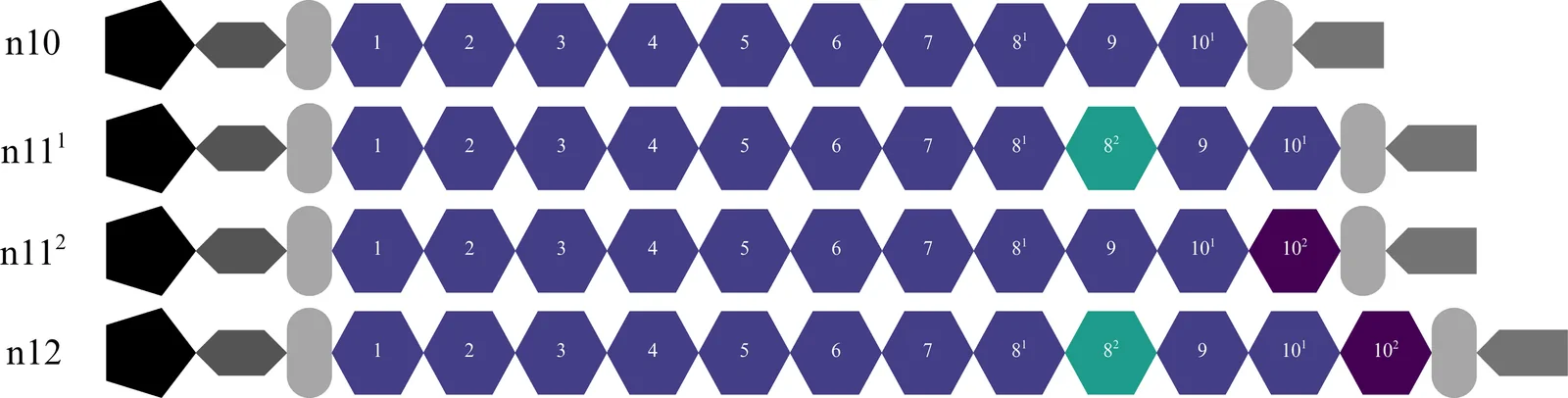
**The representation of canonical FLG alleles described in the literature.** Duplicated FLG repeats are indicated in cyan (8^*2*^) and in purple (10^*2*^).

The Oxford Nanopore sequencing platform has seen rapid improvements in accuracy in recent years, reaching a point where it can outperform short-read approaches in certain contexts (Hall et al, 2024; Sereika et al, 2022). Therefore, this technology can now characterize genes that were previously refractory to sequencing. To apply this technology in a cost-effective manner, we developed overlapping long-range PCRs coupled with long-read sequencing (Oxford Nanopore Technologies) to amplify and sequence the entire *FLG* gene. We applied this assay to 192 samples, originating from Belgium, Ireland, and Rwanda, with the goal of uncovering LOF variants that were previously unidentified.

## Results

### Belgian and Irish cohort composition and sequencing

As a proof of concept, we sequenced the Genome in a Bottle sample HG002 with Oxford Nanopore Technologies. Our custom genome, composed of *FLG* alleles with 10 repeats (from hg19) and 11 and 12 repeats (from the T2T diploid assembly generated from sample HG002 [Nurk et al, 2022]), was used as a reference for aligning the sequenced reads. As expected, no variants were detected. We then processed a local cohort composed of 29 patients with AD and 67 healthy controls. LOF variants were found to be significantly more associated with AD; 14 patients carried at least 1 variant (48.3%), compared with 8 controls (11.9%) who were heterozygous for LOF variation (Fisher’s test, *P* = .0003194, OR with 95% confidence interval = 2.39–19.89). The most frequent variants were p.Arg501∗ and c.2282_2285del, found in 34.5 and 27.6%, respectively, of samples with LOF variants (23.8 and 33.3% for patients with AD). Similarly, 2 less frequent but commonly tested variants, p.Arg2447∗ and p.Ser3247∗, were found in 6.9 and 3.5%, respectively, of our samples (both in 4.8% for patients with AD). Thus, 72.4% of the LOF variants we detected in our cohort (66.7% in patients with AD) are commonly tested for in clinical studies (Table 1). The other variants detected were made up of 8 rare LOF variants, including one—c.10472del—not previously associated with AD or observed in the Genome Aggregation Database (Chen et al, 2024) (Figures 2a and 3). More interestingly, we were able to precisely phase heterozygous variants and, therefore, determine whether a sample is a compound heterozygote, thanks to the long reads. Of the 14 patients with AD with a *FLG* LOF variant, 7 were heterozygous, 1 was homozygous for a LOF variant, whereas the remaining 6 were compound heterozygous carriers (42.9%).

**Table 1:** LOF Variants Identified in Our Cohort (n = 192)

Sample	Condition	Allele 1	LOF 1	Allele 2	LOF 2	AD Severity
BE-001	Case	n10	**c.2282_2285del**	n11^1^	p.Arg2447∗	Moderate
BE-002	Case	n11^1^	**p.Arg501∗**	n11^1^	p.Arg501∗	Moderate
BE-003	Case	n10	**c.2282_2285del**	—	—	Severe
BE-008	Case	n10	**c.2282_2285del**	n11^1^	p.Arg501∗	Ichthyosis
BE-038	Case	n10	**c.2282_2285del**	n11^1^	c.9280del	Severe
BE-039	Case	n10	**c.2282_2285del**	—	—	Moderate
BE-082	Case	n12	**p.Ser3247∗**	—	—	Unknown
BE-085	Case	n11^1^	**p.Arg501∗**	—	—	Moderate
BE-086	Case	n10	**c.2282_2285del**	n10	p.Arg3419∗	Moderate
BE-089	Case	n11^1^	p.Arg2685∗	n12	c.3254_3257del	Severe
BE-091	Case	n11^1^	**p.Arg501∗**	—	—	Severe
BE-093	Case	n10	**c.2282_2285del**	n11^1^	p.Arg2613∗	Severe
BE-094	Case	n10	p.Arg1474∗	—	—	Moderate
BE-095	Case	n12	c.10472del	—	—	Severe
IR-004	Case	n11^1^	**p.Arg501∗**	—	—	Unknown
IR-005	Case	n11^1^	**p.Arg501∗**	—	—	Unknown
IR-006	Case	n11^1^	**p.Arg501∗**	—	—	Unknown
IR-007	Case	n10	**c.2282_2285del**	—	—	Unknown
IR-008	Case	n11^1^	**p.Arg501∗**	—	—	Unknown
IR-012	Case	n12	**p.Ser3247∗**	—	—	Unknown
IR-014	Case	n10	**c.2282_2285del**	—	—	Unknown
IR-018	Case	n10	**c.2282_2285del**	—	—	Unknown
IR-019	Case	n11^1^	**p.Arg501∗**	—	—	Unknown
BE-019	Control	n11^1^	**p.Arg501∗**	—	—	—
BE-032	Control	n10	**p.Arg501∗**	—	—	—
BE-056	Control	n11^1^	**p.Arg2447∗**	—	—	—
BE-058	Control	n10	**c.2282_2285del**	—	—	—
BE-062	Control	n11^1^	**p.Arg501∗**	—	—	—
BE-072	Control	n10	p.Gly1707∗	—	—	—
BE-073	Control	n11^1^	**p.Arg501∗**	—	—	—
BE-079	Control	n11^1^	**p.Arg501∗**	—	—	—

Abbreviations: AD, atopic dermatitis; LOF, loss-of-function.

Boldface indicates common LOF variants.

**Figure 2:**
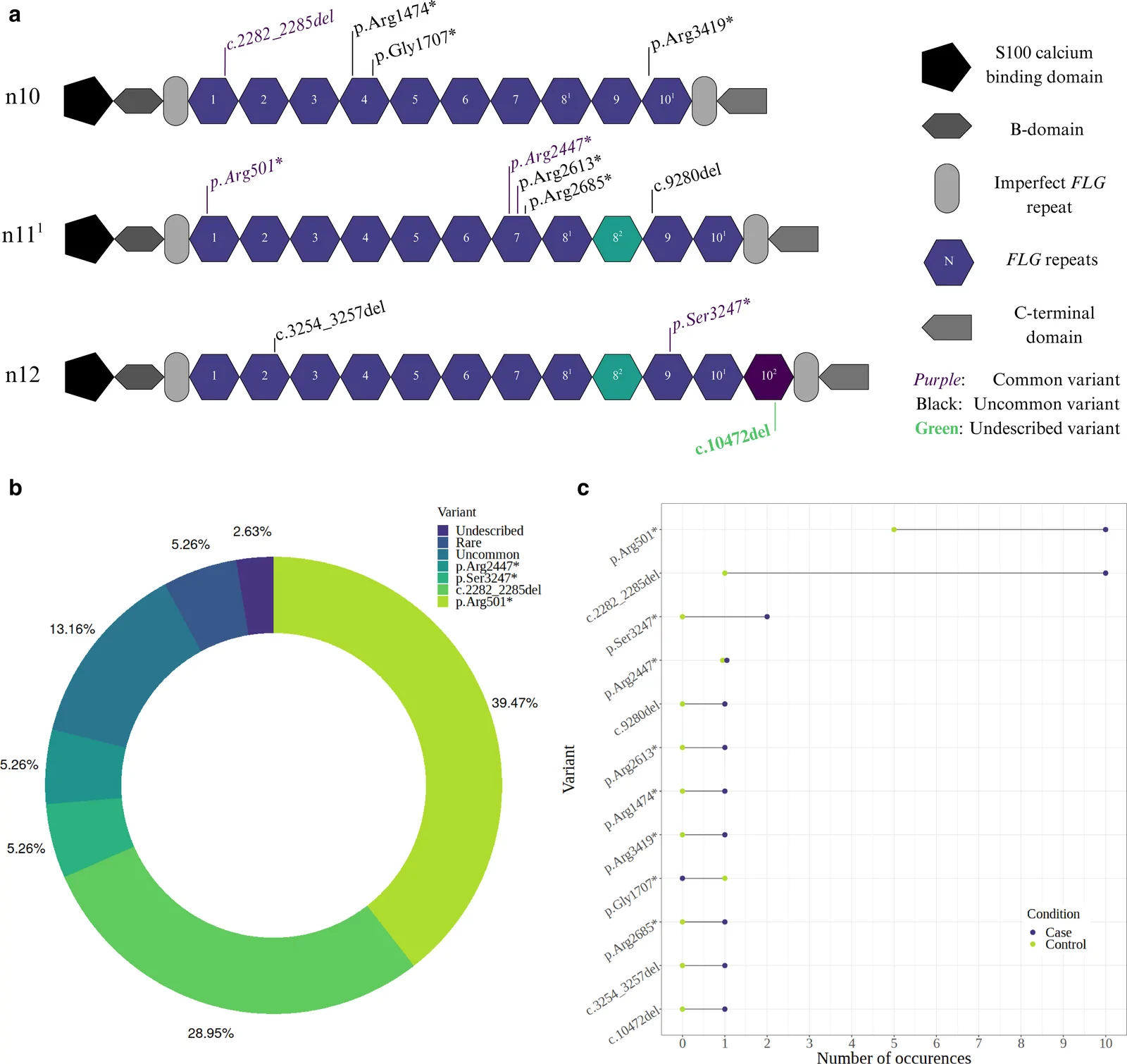
**LOF variants within our in-house cohort.** (**a**) LOF variants and the allele they were observed on. The undescribed variant we detected, c.10472del, is indicated in green and found within the duplicated 10 repetition on the 12 repeats allele. (**b**) The frequency of the 4 most common LOF variants searched in screening as well as the frequency of uncommon, rare, and undescribed variants. (**c**) The number of occurrences of each LOF variant found in our Belgian and Irish cohorts, either in our patients with AD (purple), our control cohort (green), or both. The number of occurrences of each LOF variant found in our Belgian and Irish cohorts, either in our patients with AD (purple), our control cohort (green), or both. AD, atopic dermatitis; LOF, loss-of-function.

**Figure 3:**
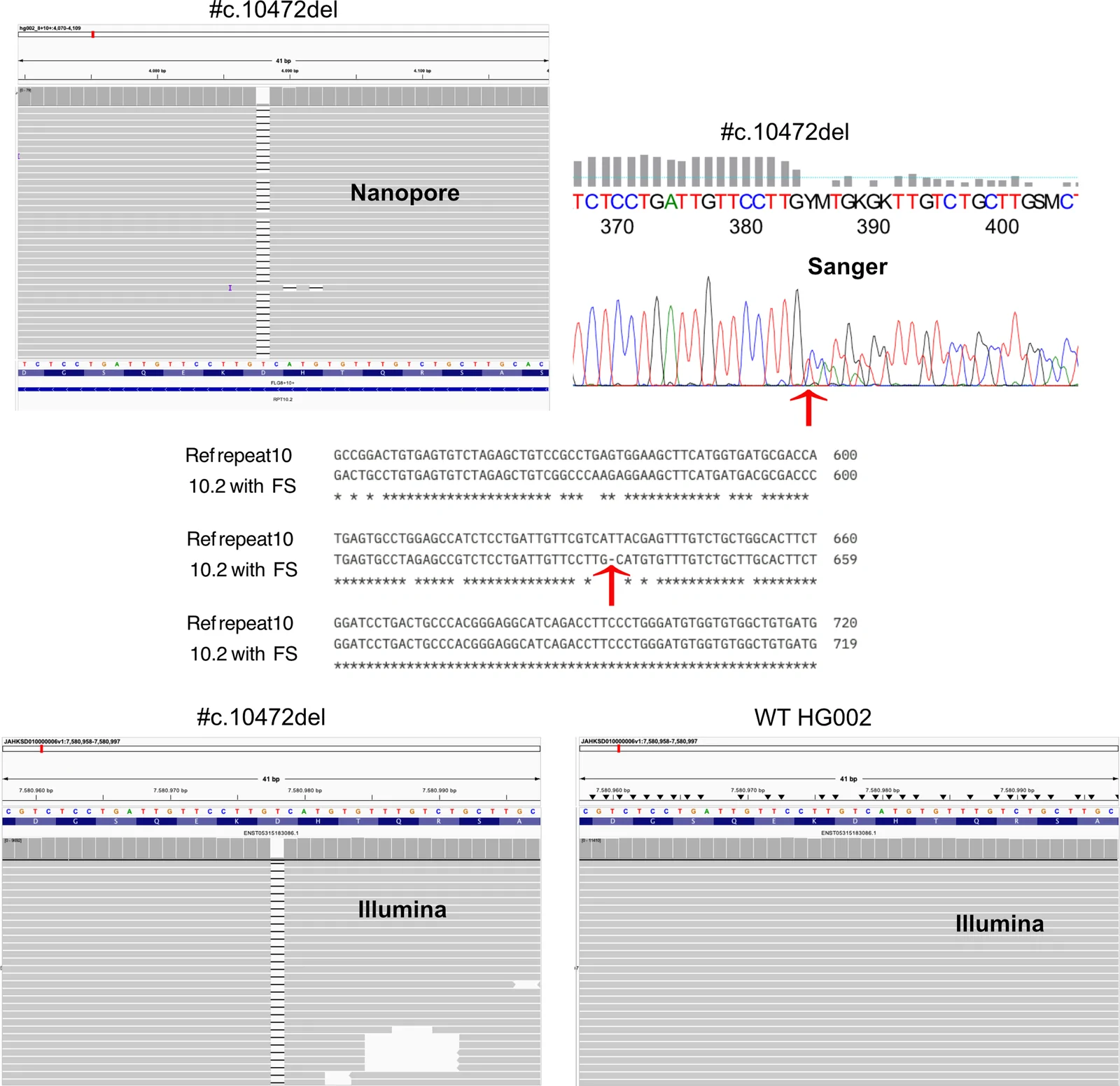
**Identification of LOF variant c.10472del.** IGV screenshot of a previously undescribed *FLG* LOF sequence variant, c.10472del. Top left: Nanopore reads are mapped to the maternal allele of the HG002-T2T assembly (n12). Top right: A shortened 800 bp PCR amplicon was designed for resequencing; the red arrow shows the position of the deletion, and the Sanger read becomes out of phase downstream. The middle panel shows a comparison of the sequence of repeat 10 in the 10-repeat allele with the second copy of repeat 10 in the 12-repeat *FLG* allele. The bottom panel shows a shorter amplicon sequenced on the Illumina platform and mapped back to the maternal allele of the HG002-T2T assembly; the HG002 genome in a bottle was also sequenced as a control. IGV, Integrative Genomics Viewer; LOF, loss-of-function.

In addition to our cohort of 96 Belgian samples, we sequenced 20 additional samples from patients with AD of Irish origin. Ten of these samples had been screened for the 4 most common LOF variants (p.Arg501∗, c.2282_2285del, p.Ar2447∗, and p.Ser3247∗), and 5 of them were found to be heterozygous carriers. We sequenced all 20 samples and were able to identify the 5 known variants. We were also able to detect 4 LOF variants in the 10 samples that had not been tested. Therefore, we found 9 LOF variants in our Irish cohort (45.0%), and once again, p.Arg501∗ and c.2282_2285del were the 2 most frequent variants (55.6 and 33.3%, respectively), with 1 sample carrying p.Ser3247∗ (11.1%). In total, 79.0% of the detected LOF variants in our cohort are commonly tested for in clinical studies (Figure 2b and c).

### Rwandan cohort sequencing

It has been known for some time that the frequency of LOF variants in *FLG* is much lower in patients with AD of sub-Saharan origin (Thawer-Esmail et al, 2014; Winge et al, 2011), and we hypothesized that a long-read approach could potentially uncover overlooked variants in these populations. To address the question, we examined a cohort composed of samples from Rwanda, in collaboration with the Center Hospitalier de Kigali, Rwanda. This cohort was composed of 76 samples, with 68 patients with AD and 8 controls. In contrast to the European patients with AD, no LOF variants were observed in the Rwandan cohort. This mirrors previous work that indicated that *FLG* LOF variants are uncommon in sub-Saharan populations (Margolis et al, 2019; Stefanovic and Irvine, 2024; Thawer-Esmail et al, 2014; Winge et al, 2011).

### Undescribed *FLG* alleles

Although we did not uncover any LOF variants in the Rwandan samples, we noticed that a large fraction carried previously undescribed *FLG* alleles that did not match any of the 4 canonical *FLG* alleles (Eaaswarkhanth et al, 2016). In addition, however, at a lower frequency, we also observed a number of undescribed alleles in our European cohort. First, we identified a subset of samples that exhibit large insertions and deletions when compared with known *FLG* alleles. These insertions/deletions corresponded to the ∼975bp *FLG* repeat or a multiple of it. We observed 1 allele with 9 *FLG* repeats, 1 with 13 *FLG* repeats, and 1 with 16 (Figures 4a and Figure 5, Figure 6, Figure 7 and Table 2). We confirmed the number of *FLG* repeats in samples carrying 16 repeats by nanopore adaptive sampling by targeting the *FLG* gene and its flanking region. Moreover, we found multiple samples carrying alleles that fell within the normal range for the number of *FLG* repeats but showed regions of high SNP density (∼75 single nucleotide variation per kb, covering ∼500 bp to ∼2.2 kb) when mapped to 1 of the known alleles carrying the same number of *FLG* repeats (Figures 4a, and Figure 6, Figure 7, Figure 8, Figure 9, Figure 10, Figure 11). Alleles with 9, 13, and 16 repeats were confirmed using agarose gel electrophoresis (Figure 12). Careful examination of the reads and consensus sequence generated from each sample indicated that these undescribed alleles originate from intragenic recombination among the *FLG* repeats, resulting in the addition or subtraction of 1 *FLG* repeat (mostly concentrated in the 3′ end of exon 3) and point to extensively unrecognized diversity in *FLG* exon 3 (Table 3).

**Figure 4:**
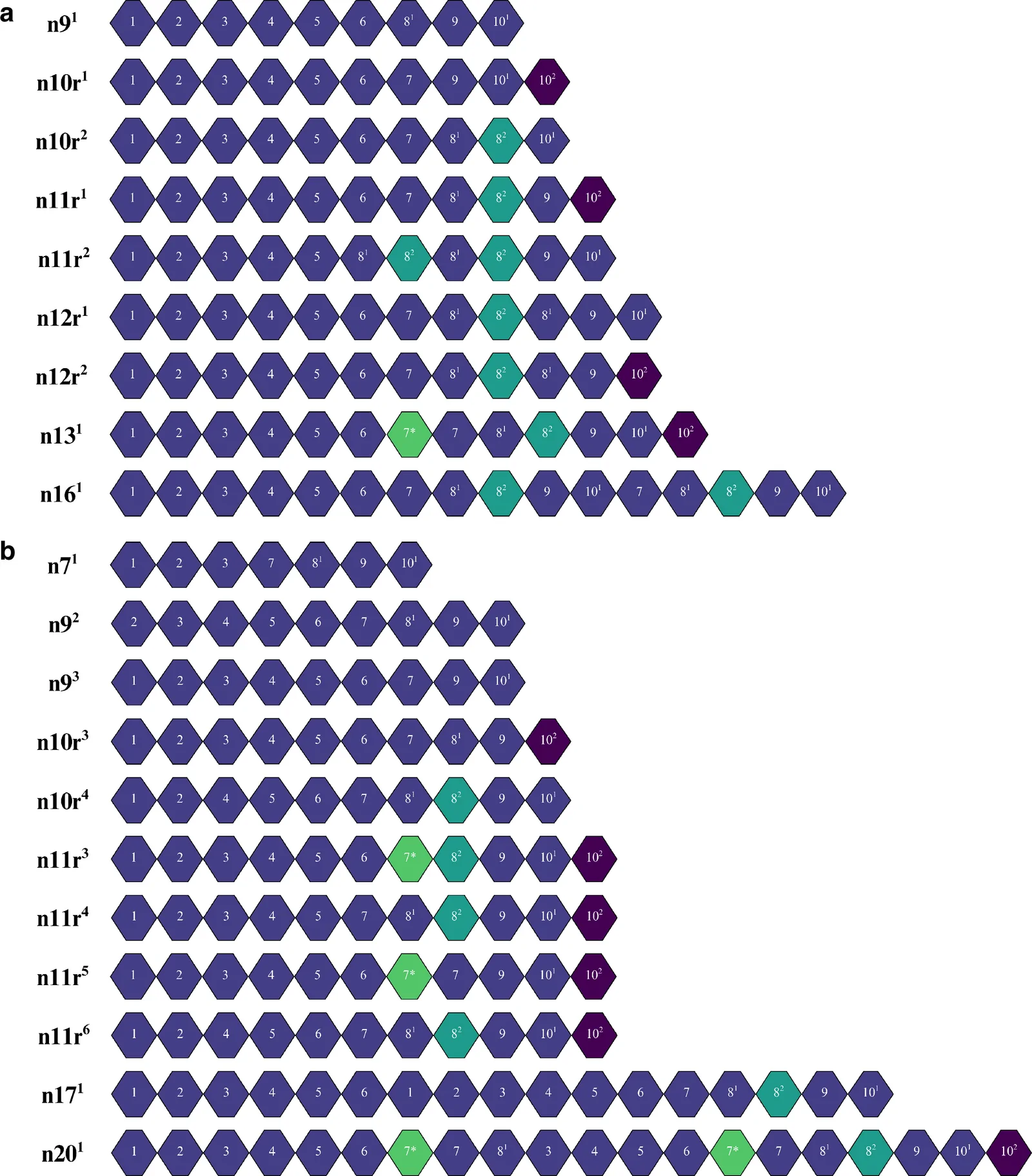
**Previously undescribed *FLG* alleles.** (**a**) Representation of previously undescribed *FLG* alleles found within our in-house cohort. The green repetition corresponds to a duplication of the 7^th^*FLG* repeat not described in the literature before. (**b**) Representation of previously undescribed *FLG* alleles found in the 1000 Genome project public data.

**Figure 5:**
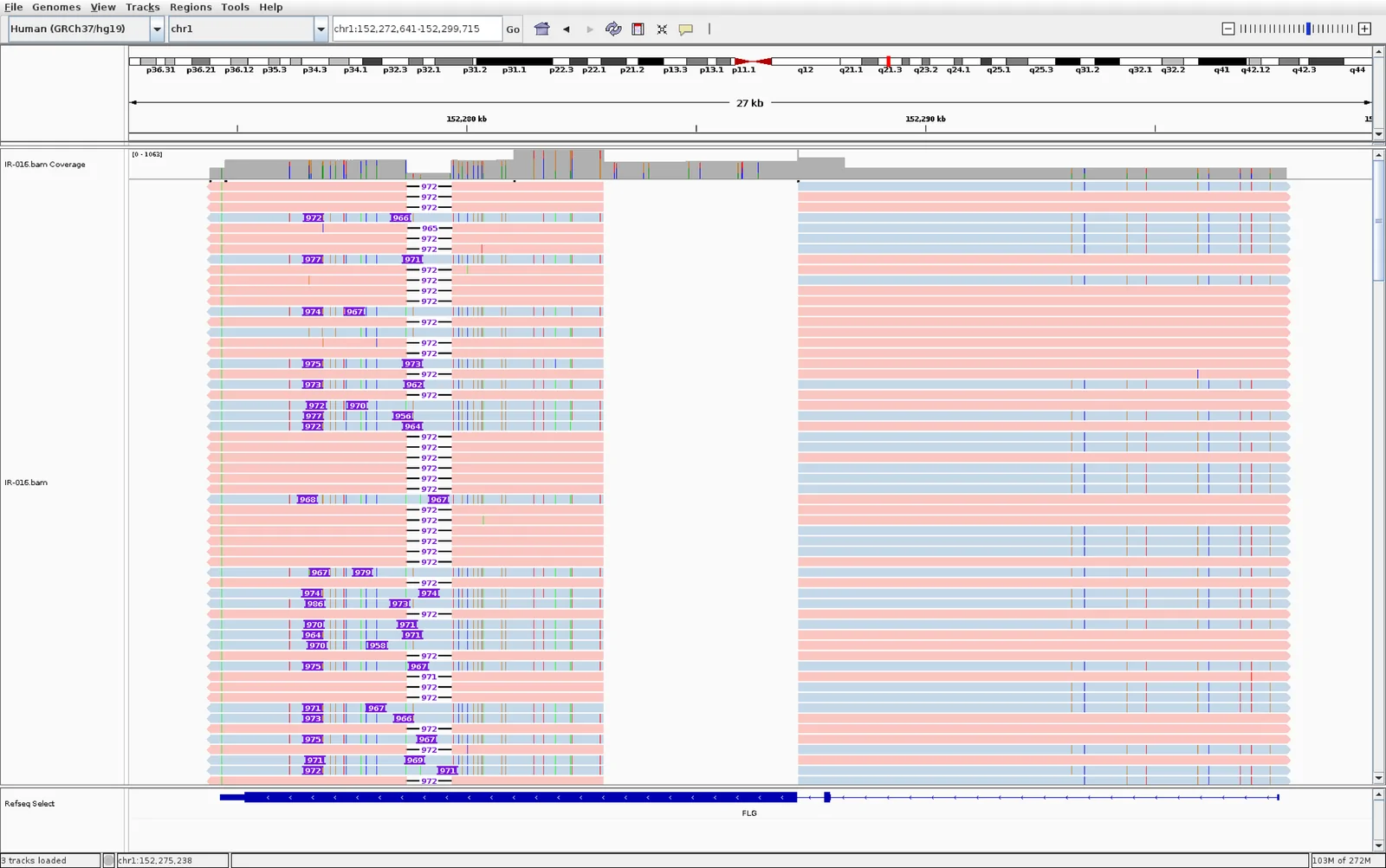
**Visualization of undescribed FLG allele n9^1^.** IGV screenshot of an undescribed *FLG* allele n9^1^ of sample IR-016 is shown. Reads were mapped to the hg19 reference genome. Reads with n9^1^ information are colored in red and show a deletion of ∼972 bp (white gap). IGV, Integrative Genomics Viewer.

**Figure 6:**
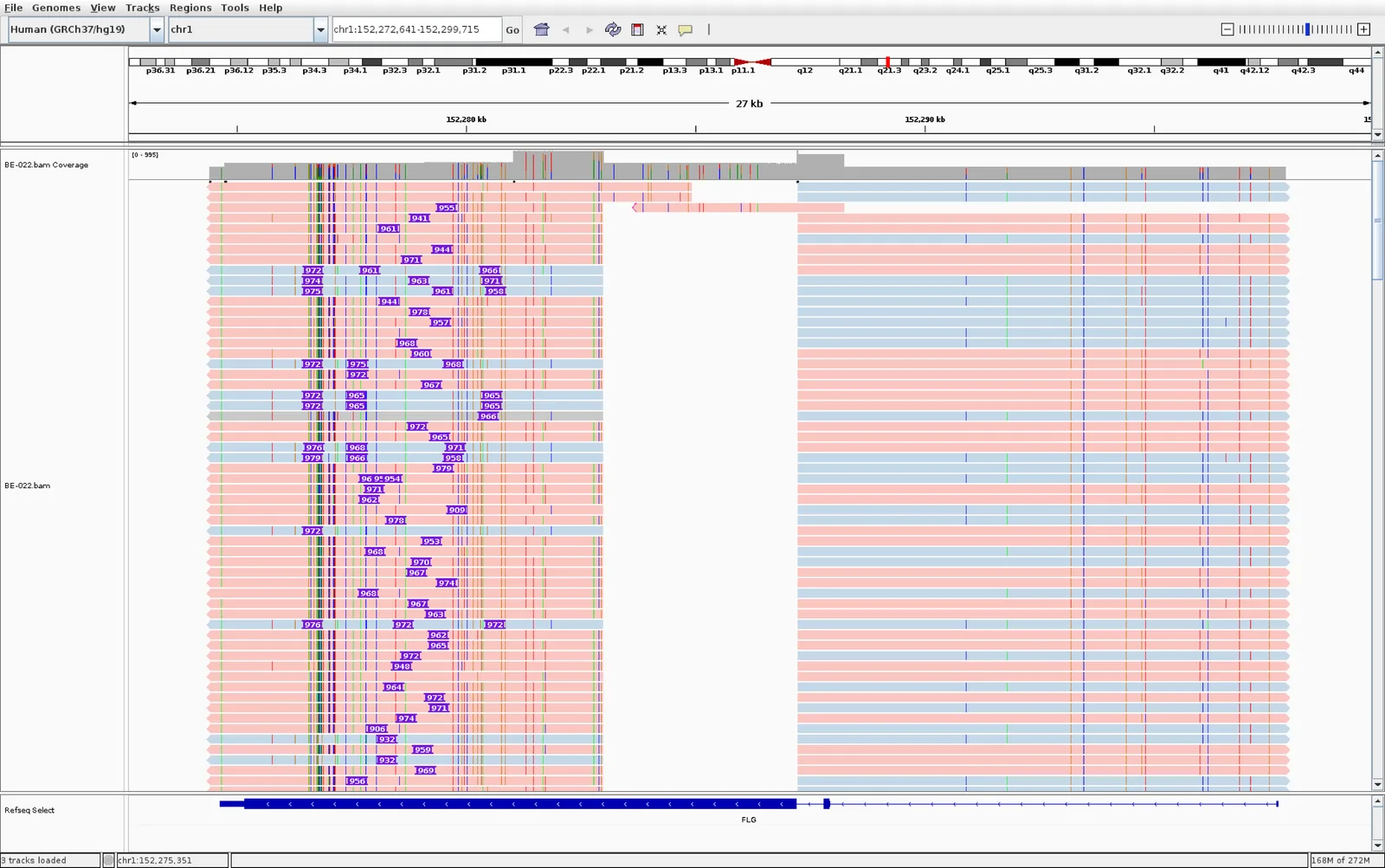
**Visualization of undescribed FLG alleles n11r^1^ and n13^1^.** Shown is the IGV screenshot of undescribed *FLG* alleles n11r^1^ and n13^1^ of sample BE-022. Reads were mapped to the hg19 reference genome. Reads with n11r^1^ information are colored in red, with an insertion of ∼972 bp (purple rectangle) and a high SNP density in the 3′ end of exon 3 (vertical-colored lines). Those with n13^1^ information are colored in blue, with 3 insertions of ∼972 bp each. IGV, Integrative Genomics Viewer.

**Figure 7:**
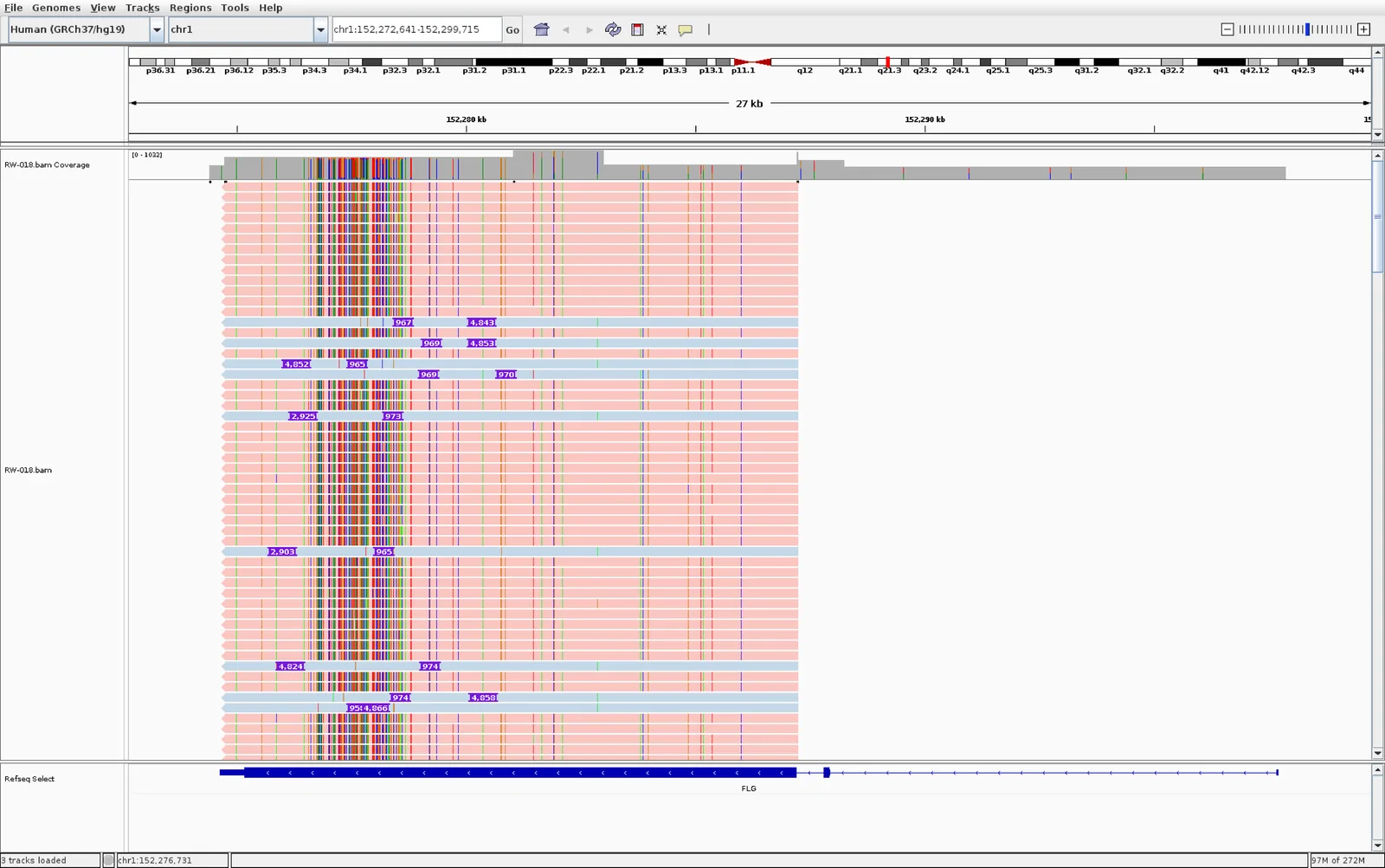
**Visualization of undescribed FLG alleles n10r^1^ and n16^1^.** Shown is the IGV screenshot of undescribed *FLG* alleles n10r^1^ and n16^1^ of sample RW-018. Reads were mapped to the hg19 reference genome. Reads with n10r^1^ information are colored in red, with a high SNP density (vertical-colored lines) at the 3′ end of exon 3. Those with n16^1^ information are colored in blue, with multiple insertions, one of ∼972 bp and the other of ∼4860 bp (purple rectangle). IGV, Integrative Genomics Viewer.

**Table 2:** Description of Undescribed Alleles in Rwandan Samples

Allele	Allele of Origin	Number of Samples	Number of Haplotype	Description
n9^1^	n10	2	2	Deletion of repeat 7
n10r^1^	n12	42	50	Deletion of repeats 8^1^ and 8^2^
n12r^1^	n11	4	4	Insertion of 1 repeat 8^1^ between repeats 8 and 9
n13^1^	n12	5	5	Insertion of 1 repeat 7∗ between repeats 6 and 7
n16^1^	n11	1	1	Insertion of 5 repeats (7-8^1^-8^2^-9-10^1^) between repeats 6 and 7

**Figure 8:**
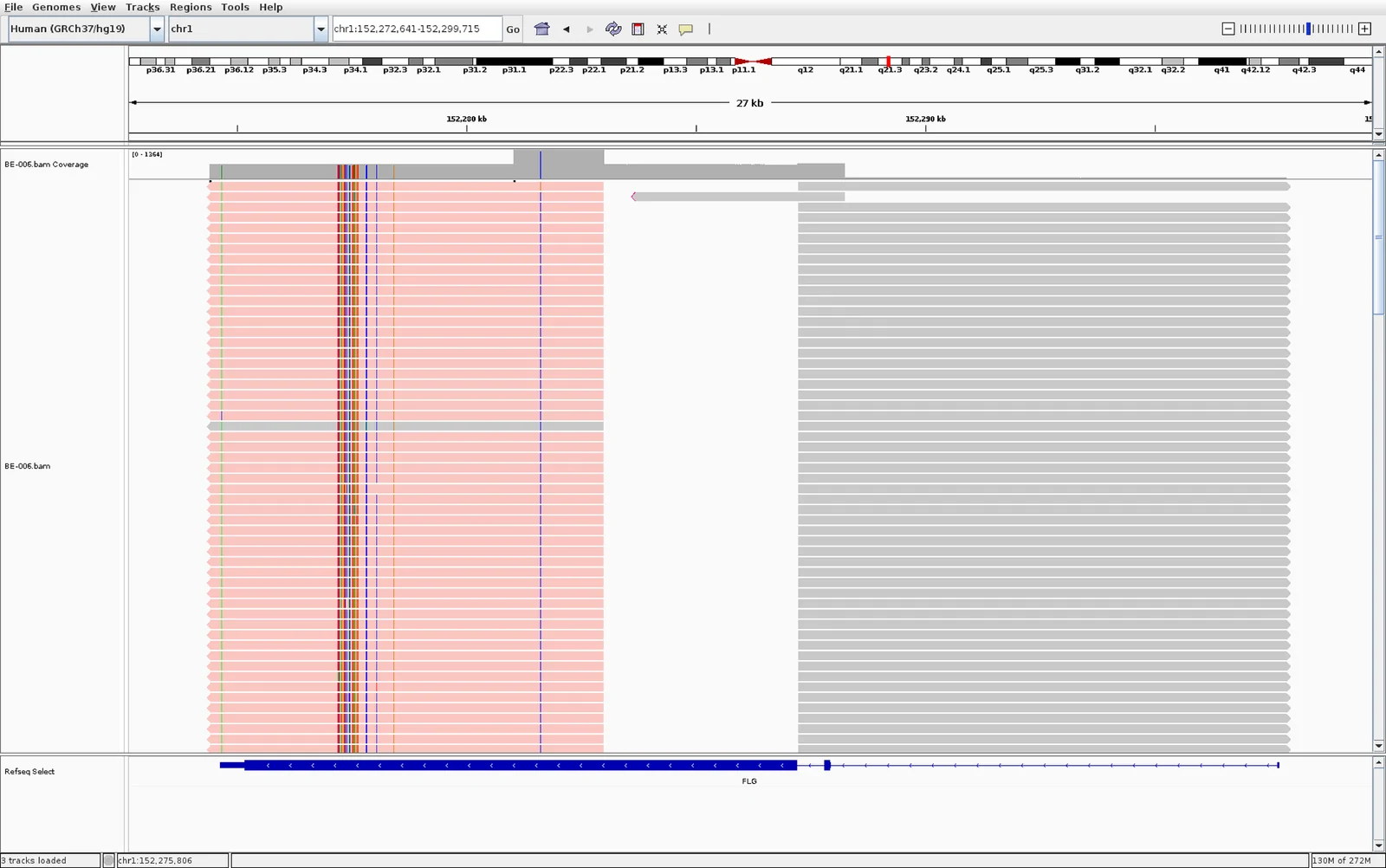
**Visualization of undescribed FLG allele n10r^2^.** Shown is the IGV screenshot of the undescribed *FLG* allele n10r^2^ of sample BE-006. Reads were mapped to the hg19 reference genome. Reads with n10r^2^ information are colored in red, with a high SNP density (vertical-colored lines) at the 3′ end of exon 3. IGV, Integrative Genomics Viewer.

**Figure 9:**
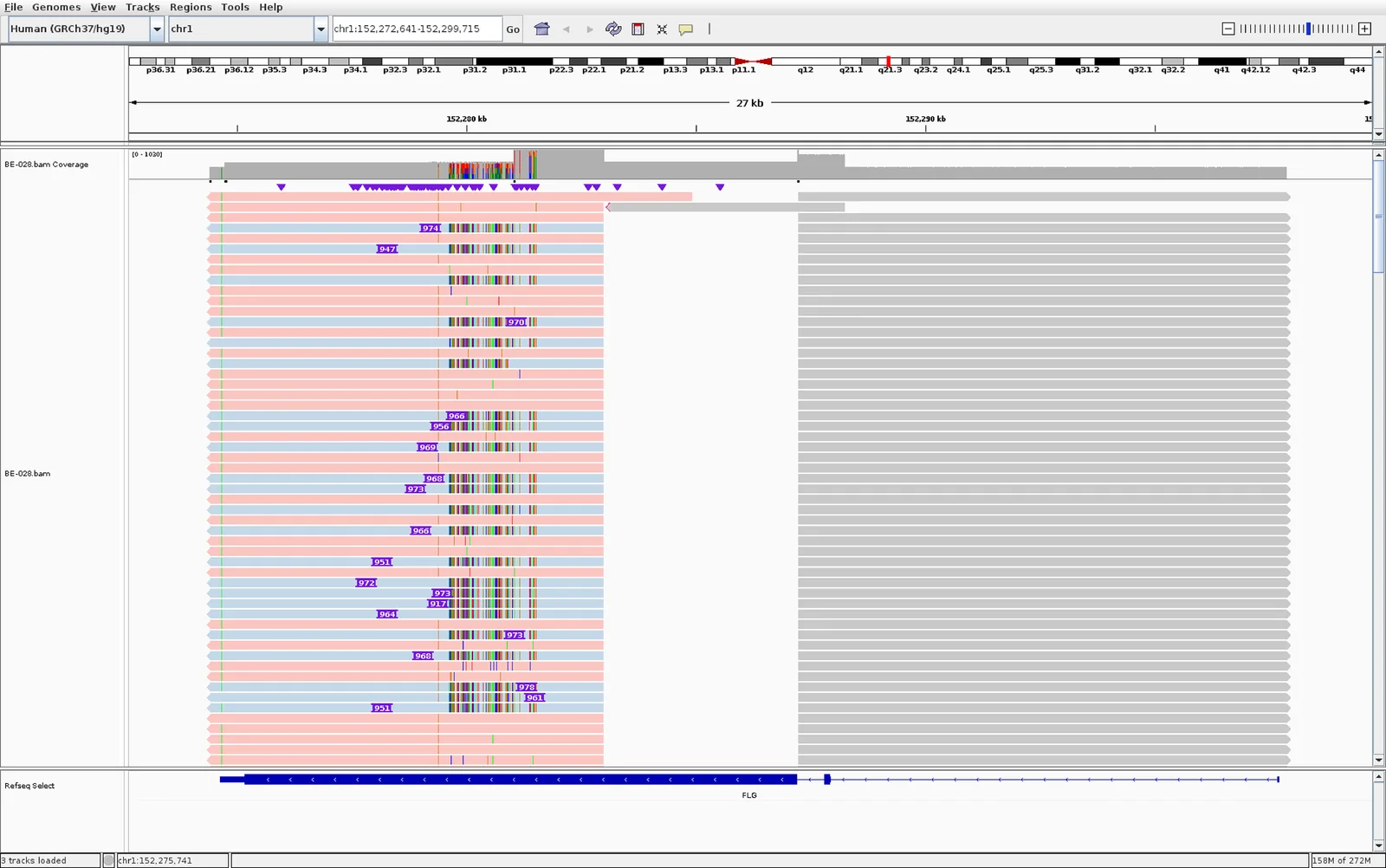
**Visualization of undescribed FLG allele n11r^2^.** Shown is the IGV screenshot of the undescribed *FLG* allele n11r^2^ of sample BE-028. Reads were mapped to the hg19 reference genome. Reads with n11r^2^ information are colored in blue and show an insertion of ∼972 bp (purple rectangle) and a high SNP density (vertical-colored lines) in the middle of exon 3. IGV, Integrative Genomics Viewer.

**Figure 10:**
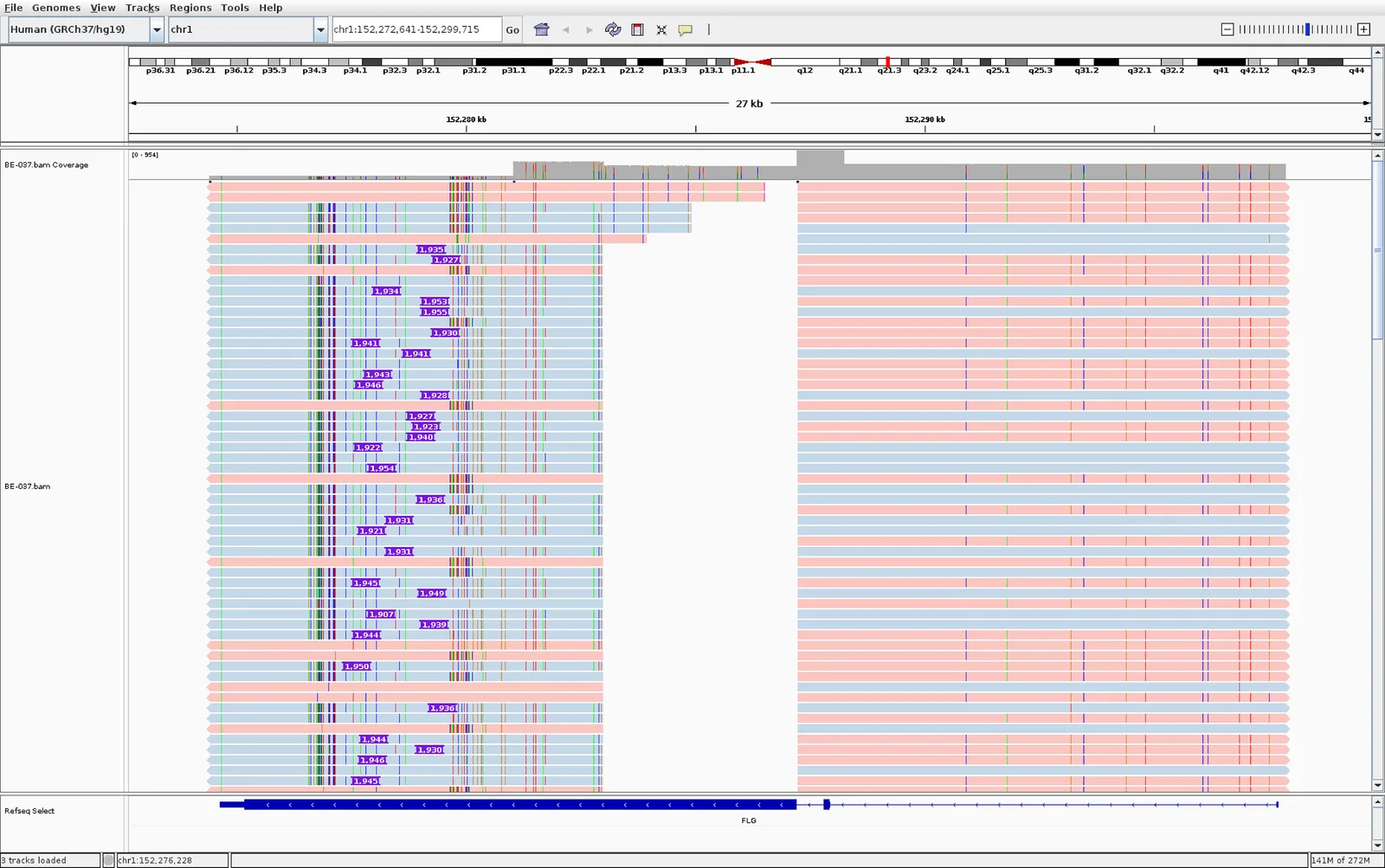
**Visualization of undescribed FLG allele n12r^2^.** Shown is the IGV screenshot of the undescribed *FLG* allele n12r^2^ of sample BE-037. Reads were mapped to the hg19 reference genome. Reads with n12r^2^ information are colored in blue and show an insertion of ∼1944 bp (purple rectangle) and a high SNP density (vertical-colored lines) at the 3′ end of exon 3. IGV, Integrative Genomics Viewer.

**Figure 11:**
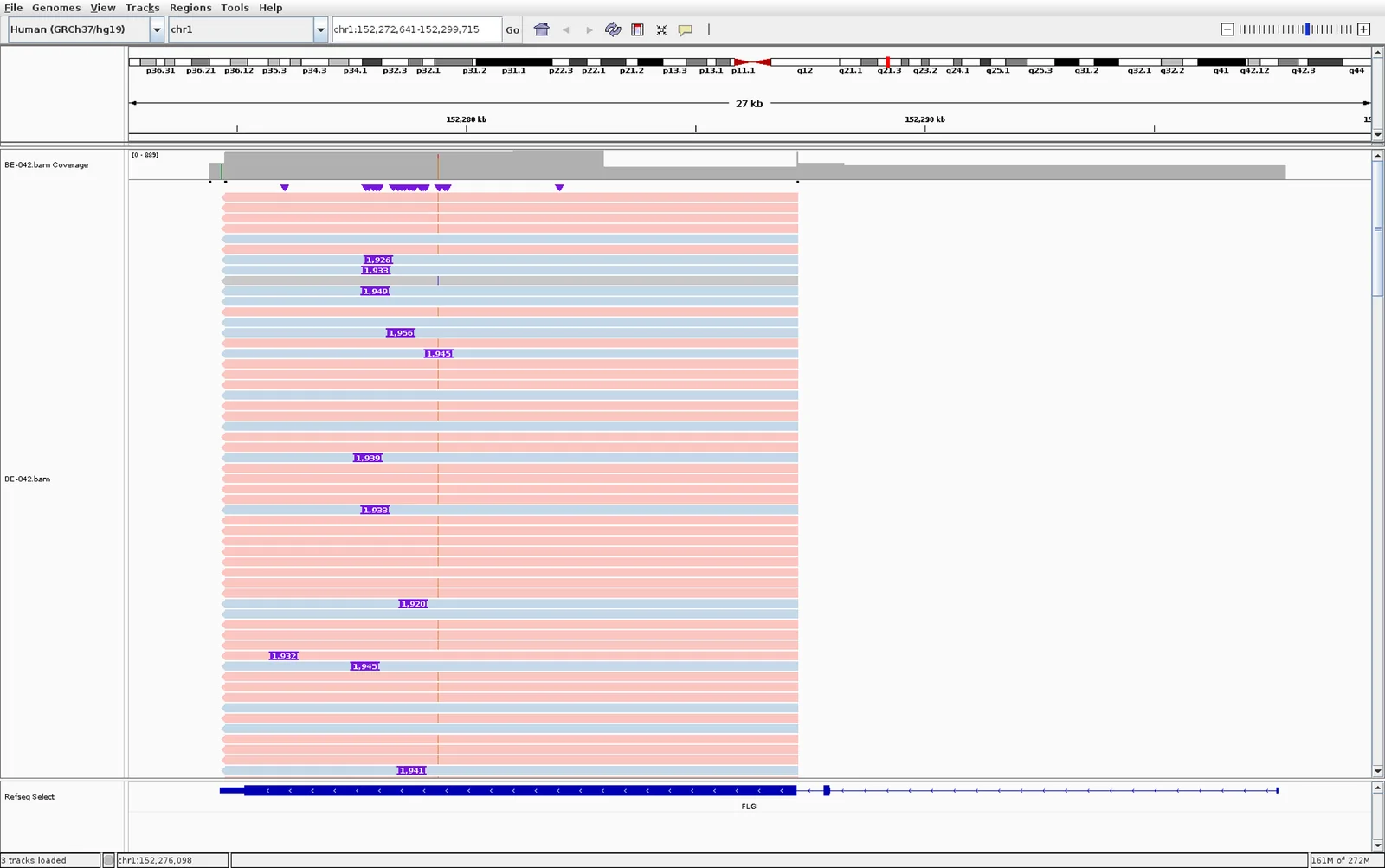
**Visualization of undescribed FLG allele n12r^1^.** Shown is the IGV screenshot of the undescribed *FLG* allele n12r^1^ of sample BE-042. Reads were mapped to the hg19 reference genome. Reads with n12r^1^ information are colored blue, with an insertion of ∼1944 bp (purple rectangle). IGV, Integrative Genomics Viewer.

**Figure 12:**
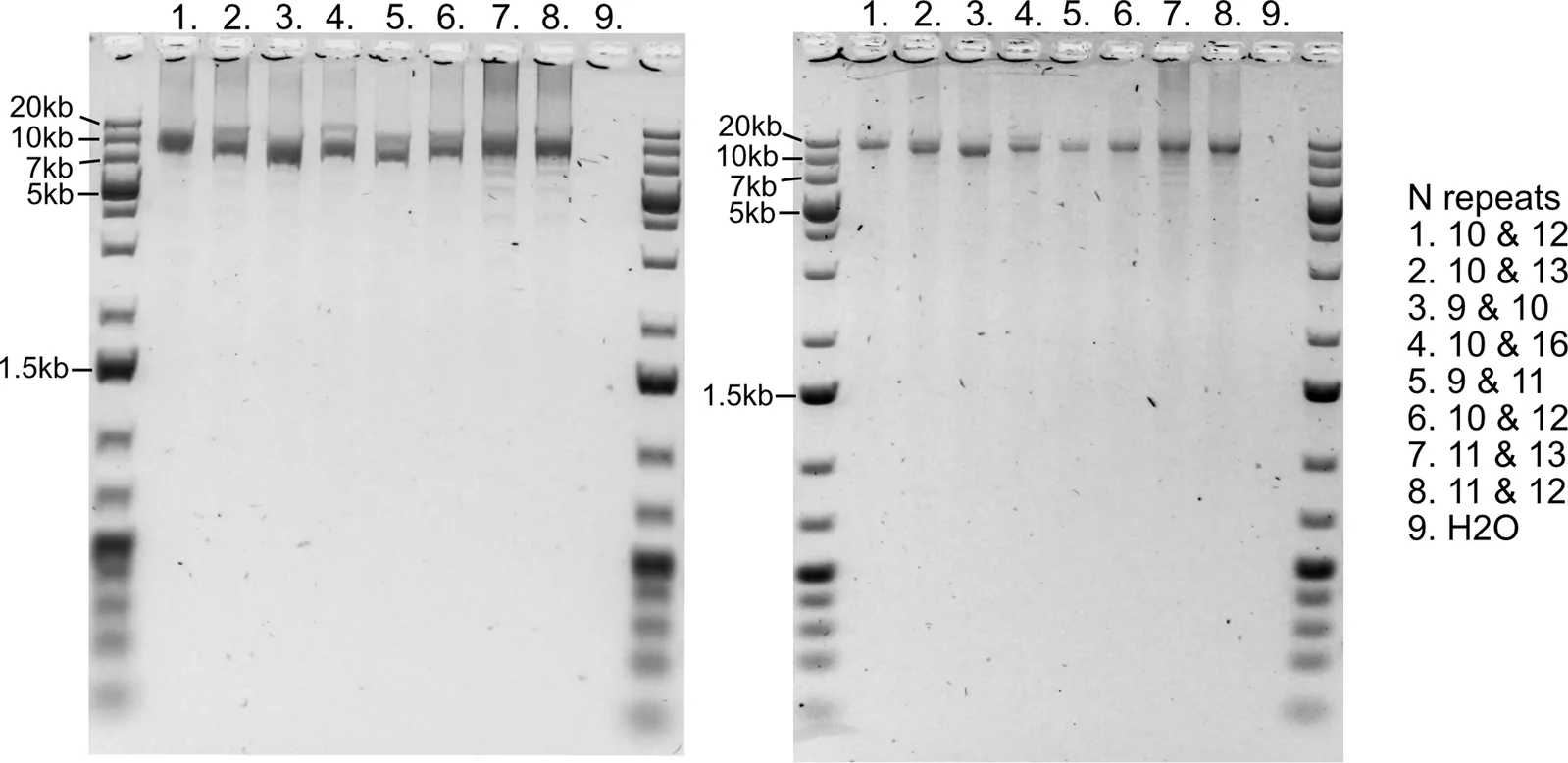
**A 1% agarose gel, 8 samples carrying alleles of different lengths.** On the left is shown the F3 amplicon, which amplifies the 3′ end of exon 3 and has an expected product size of 8.5 kb for the 10-repeat allele. The right shows the F4 amplicon, which amplifies all of exon 3, generating a PCR product of 12.5 kb for the 10-repeat allele. When the 2 alleles are relatively close in size (∼ 2 kb), it is generally not possible to resolve them; however, the 9-repeat allele bands are clearly shorter (3 and 5). In addition, in samples 2 and 4, where the alleles differ by ∼3 kb and ∼6 kb, respectively, the 2 bands are apparent for the F3 amplicons. For the F4 amplicon in sample 4, 2 PCR bands are observed, although the larger is less intense than the lower.

**Table 3:** Description of Undescribed Alleles in Belgian and Irish Samples

Allele	Allele of Origin	Number of Samples	Number of Haplotype	Description
n9^1^	n10	1	1	Deletion of repeat 7
n10r^1^	n12	5	5	Deletion of repeats 8^1^ and 8^2^
n10r^2^	n11	1	1	Deletion of repeat 9
n11r^1^	n12	4	4	Deletion of repeat 10^1^
n11r^2^	n12	1	1	Deletion of repeats 6 and 7 and insertion of 1 repeat 8^1^ and 1 repeat 8^2^ between repeats 5 and 8^1^
n12r^1^	n11	3	3	Insertion of 1 repeat 8^1^ between repeats 8^2^ and 9
n12r^2^	n12	1	1	Insertion of 1 repeat 8^1^ between repeats 8^2^ and 9 and deletion of repeat 10^1^
n13^1^	n12	1	1	Insertion of 1 repeat 7∗ between repeats 6 and 7

### Public sample analysis

To exclude the possibility that technical artifacts in our approach could have generated these undescribed alleles, we examined the publicly available whole-genome sequencing long-read data from the 1000 Genomes Project (Gustafson et al, 2024; Schloissnig et al, 2025; Auton et al, 2015). We processed long-read sequencing data from 1134 samples, with 23 sequenced by Pacific Biosciences HiFi sequencing and 1111 by Oxford Nanopore Technologies sequencing. For each of the 1134 samples, reads overlapping *FLG* were extracted, and we kept only samples with at least 2 reads covering the whole exon 3. We were able to keep 1010 samples in total, with the following origin repartition: 279 from African/African-descent populations (27.6%), 163 from South American populations (16.1%), 192 from East Asian populations (19.1%), 191 from South Asian/South Asian–descent populations (18.9%), and 185 from European/European-descent populations (18.3%).

We then mapped these samples to the known *FLG* haplotypes containing 10, 11, and 12 repeats. We checked for and reported any allele with unusual CNVs. We found a total of 8 undescribed alleles with a number of repeats that differed from the 4 established alleles (Table 4). We were also able to distinguish 11 alleles with 10, 11, and 12 repeats whose sequences, when aligned to the canonical allele of the same length, exhibited a large density of SNPs around 1 or 2 repeats (Table 4). In total, we discovered 20 undescribed *FLG* alleles, in addition to observing the 4 canonical *FLG* alleles (Figure 4). Of particular note, in the public data, we identified a remarkable range of lengths for exon 3, with the number of *FLG* repeats observed ranging from 7 to 20. In addition, we confirmed the structure of 1000 genome samples carrying 7, 17, and 20 repeats by carrying out nanopore adaptive sampling targeting the *FLG* and flanking region (Figure 4b). In addition, all but 3 undescribed haplotypes observed in our cohort were also observed in the public database. Only the n11r^2^, n12r^2^, and n16^1^ alleles were observed only in our cohort.

**Table 4:** Count of all *FLG* Alleles Depending on Sample Origins

Allele	Africa	Europe	East Asia	South Asia	South America
n10	123	181	79	57	45
n11^1^	173	325	53	124	131
n11^2^	0	1	23	4	1
n12	39	64	215	167	130
*n7*^*1*^	0	0	1	0	0
*n9*^*1*^	41	1	0	0	0
*n9*^*2*^	6	0	0	0	0
*n9*^*3*^	1	1	0	0	0
*n10r*^*1*^	275	4	4	0	7
*n10r*^*2*^	1	4	0	3	0
*n10r*^*3*^	1	0	0	0	0
*n10r*^*4*^	1	0	0	0	0
*n11r*^*1*^	0	13	8	26	8
*n11r*^*2*^	0	1	0	0	0
*n11r*^*3*^	1	0	0	0	0
*n11r*^*4*^	0	0	1	0	0
*n11r*^*5*^	4	0	0	0	0
*n11r*^*6*^	0	0	0	0	1
*n12r*^*1*^	10	4	0	1	1
*n12r*^*2*^	0	1	0	0	0
*n13*^*1*^	33	1	0	0	1
*n16*^*1*^	1	0	0	0	0
*n17*^*1*^	0	1	0	0	0
*n20*^*1*^	0	0	0	0	1

Previously undescribed alleles are italicized.

### *FLG* allele frequencies

We then counted the number of *FLG* alleles in all our cohorts (Table 5) as well as the different genotypes (Table 6). To gain a better understanding of the distribution of alleles in different populations, we checked the frequency of all *FLG* alleles. When taking into account both known alleles and those with an unusual number of repeats, the most frequent allele for each population was n10 for Africa (56.5%), n11^1^ for Europe (56.3%) and South America (42.9%), and n12 for East Asia (56.0%) and South Asia (44.0%) (Figure 13). The frequencies of alleles with unusual CNVs are 11.5, 0.7, 0.6, 0.3, and 0.0% for Africa, Europe, South America, East Asia, and South Asia, respectively (Figure 14). However, when taking into account undescribed alleles with 10, 11, and 12 repeats, the frequencies became the following: n10r^1^ for Africa (38.7%), n11^1^ for Europe (54.0%) and South America (40.2%), and n12 for East Asia (56.0%) and South Asia (43.7%) (Figure 15). Overall, the frequencies of undescribed *FLG* alleles were 52.8, 5.1, 5.8, 3.6, and 7.9% for Africa, Europe, South America, East Asia, and South Asia, respectively (Figure 16).

**Table 5:** Description of Undescribed Alleles Found in the Public 1000 Genome Project Database

Allele	Allele of Origin	Number of Samples	Number of Haplotype	Description
n7^1^	n10	1	1	Deletion of repeats 4 to 6
n9^1^	n10	35	38	Deletion of repeat 7
n9^2^	n10	6	6	Deletion of repeat 1
n9^3^	n10	2	2	Deletion of repeat 8
n10r^1^	n12	180	236	Deletion of repeats 8^1^ and 8^2^
n10r^2^	n11	6	7	Deletion of repeat 9
n10r^3^	n12	1	1	Deletion of repeats 8^1^ and 10^1^
n10r^4^	n11	1	1	Deletion of repeat 3
n11r^1^	n12	51	51	Deletion of repeat 10^1^
n11r^3^	n12	1	1	Deletion of repeats 7 and 8^1^ and insertion of 1 repeat 7∗ between repeats 6 and 8^2^
n11r^4^	n12	1	1	Deletion of repeat 6
n11r^5^	n12	4	4	Deletion of repeats 8^1^ and 8^2^ and insertion of 1 repeat 7∗ between repeats 6 and 7
n11r^6^	n12	1	1	Deletion of repeat 3
n12r^1^	n11	10	10	Insertion of 1 repeat 8^1^ between repeats 8^2^ and 9
n13^1^	n12	28	29	Insertion of 1 repeat 7∗ between repeats 6 and 7
n17^1^	n11	1	1	Insertion of 6 repeats (1-2-3-4-5-6) between repeats 6 and 7
n20^1^	n12	1	1	Insertion of 8 repeats (7∗-7-8^1^-3-4-5-6-7∗) between repeats 6 and 7

**Table 6:** Count and Frequency of all Genotypes across all Populations

Haplotype 1	Haplotype 2	Count	Frequency	Count Africa	Frequency Africa	Count East Asia	Frequency East Asia	Count South Asia	Frequency South Asia	Count South America	Frequency South America	Count Europe	Frequency Europe
n10	n10	61	5,1	18	5,1	6	3,1	6	3,1	2	1,2	29	9,6
n10	n11^1^	161	13,4	24	6,8	11	5,7	15	7,9	18	11	93	30,9
n10	n11^2^	6	0,5	0	0	3	1,6	2	1	0	0	1	0,3
n10	n12	114	9,5	8	2,3	50	26	19	9,9	16	9,8	21	7
n10	n7^1^	1	0,1	0	0	1	0,5	0	0	0	0	0	0
n10	n9^1^	10	0,8	10	2,8	0	0	0	0	0	0	0	0
n10	n9^2^	1	0,1	1	0,3	0	0	0	0	0	0	0	0
n10	n10r^1^	41	3,4	37	10,4	0	0	0	0	2	1,2	2	0,7
n10	n10r^3^	1	0,1	1	0,3	0	0	0	0	0	0	0	0
n10	n11r^1^	18	1,5	0	0	2	1	9	4,7	4	2,5	3	1
n10	n11r^2^	1	0,1	0	0	0	0	0	0	0	0	1	0,3
n10	n11r^3^	1	0,1	1	0,3	0	0	0	0	0	0	0	0
n10	n12r^1^	2	0,2	1	0,3	0	0	0	0	0	0	1	0,3
n10	n13^1^	5	0,4	4	1,1	0	0	0	0	1	0,6	0	0
n10	n17^1^	1	0,1	0	0	0	0	0	0	0	0	1	0,3
n11^1^	n11^1^	175	14,6	22	6,2	4	2,1	27	14,1	29	17,8	93	30,9
n11^1^	n11^2^	6	0,5	0	0	5	2,6	1	0,5	0	0	0	0
n11^1^	n12	155	12,9	7	2	27	14,1	45	23,6	47	28,8	29	9,6
n11^1^	n9^1^	10	0,8	10	2,8	0	0	0	0	0	0	0	0
n11^1^	n9^2^	2	0,2	2	0,6	0	0	0	0	0	0	0	0
n11^1^	n9^3^	1	0,1	0	0	0	0	0	0	0	0	1	0,3
n11^1^	n10r^1^	71	5,9	66	18,6	0	0	0	0	3	1,8	2	0,7
n11^1^	n10r^2^	3	0,2	1	0,3	0	0	1	0,5	0	0	1	0,3
n11^1^	n11r^1^	21	1,7	0	0	2	1	7	3,7	3	1,8	9	3
n11^1^	n11r^5^	1	0,1	1	0,3	0	0	0	0	0	0	0	0
n11^1^	n12r^1^	9	0,7	4	1,1	0	0	1	0,5	1	0,6	2	0,7
n11^1^	n12r^2^	1	0,1	0	0	0	0	0	0	0	0	1	0,3
n11^1^	n13^1^	14	1,2	14	3,9	0	0	0	0	0	0	1	0,3
n11^1^	n20^1^	1	0,1	0	0	0	0	0	0	1	0,6	0	0
n11^2^	n11^2^	1	0,1	0	0	1	0,5	0	0	0	0	0	0
n11^2^	n12	14	1,2	0	0	12	6,3	1	0,5	1	0,6	0	0
n11^2^	n10r^1^	1	0,1	0	0	1	0,5	0	0	0	0	0	0
n12	n12	144	12	3	0,8	59	30,7	45	23,6	31	19	6	2
n12	n9^1^	2	0,2	1	0,3	0	0	0	0	0	0	1	0,3
n12	n9^2^	1	0,1	1	0,3	0	0	0	0	0	0	0	0
n12	n10r^1^	20	1,7	15	4,2	3	1,6	0	0	2	1,2	0	0
n12	n10r^2^	3	0,2	0	0	0	0	2	1	0	0	1	0,3
n12	n11r^1^	15	1,2	0	0	4	2,1	10	5,2	1	0,6	0	0
n12	n11r^5^	1	0,1	0	0	1	0,5	0	0	0	0	0	0
n12	n11r^6^	1	0,1	0	0	0	0	0	0	1	0,6	0	0
n12	n13^1^	1	0,1	1	0,3	0	0	0	0	0	0	0	0
n9^1^	n9^1^	3	0,2	3	0,8	0	0	0	0	0	0	0	0
n9^1^	n9^2^	2	0,2	2	0,6	0	0	0	0	0	0	0	0
n9^1^	n10r^1^	10	0,8	10	2,8	0	0	0	0	0	0	0	0
n9^1^	n10r^4^	1	0,1	1	0,3	0	0	0	0	0	0	0	0
n9^1^	n13^1^	1	0,1	1	0,3	0	0	0	0	0	0	0	0
n9^3^	n10r^1^	1	0,1	1	0,3	0	0	0	0	0	0	0	0
n10r^1^	n10r^1^	64	5,3	64	18	0	0	0	0	0	0	0	0
n10r^1^	n11r^5^	3	0,2	3	0,8	0	0	0	0	0	0	0	0
n10r^1^	n12r^1^	4	0,3	4	1,1	0	0	0	0	0	0	0	0
n10r^1^	n13^1^	10	0,8	10	2,8	0	0	0	0	0	0	0	0
n10r^1^	n16^1^	1	0,1	1	0,3	0	0	0	0	0	0	0	0
n10r^2^	n10r^2^	1	0,1	0	0	0	0	0	0	0	0	1	0,3
n11r^1^	n13^1^	1	0,1	0	0	0	0	0	0	0	0	1	0,3
n12r^1^	n13^1^	1	0,1	1	0,3	0	0	0	0	0	0	0	0
n13^1^	n13^1^	1	0,1	1	0,3	0	0	0	0	0	0	0	0

**Figure 13:**
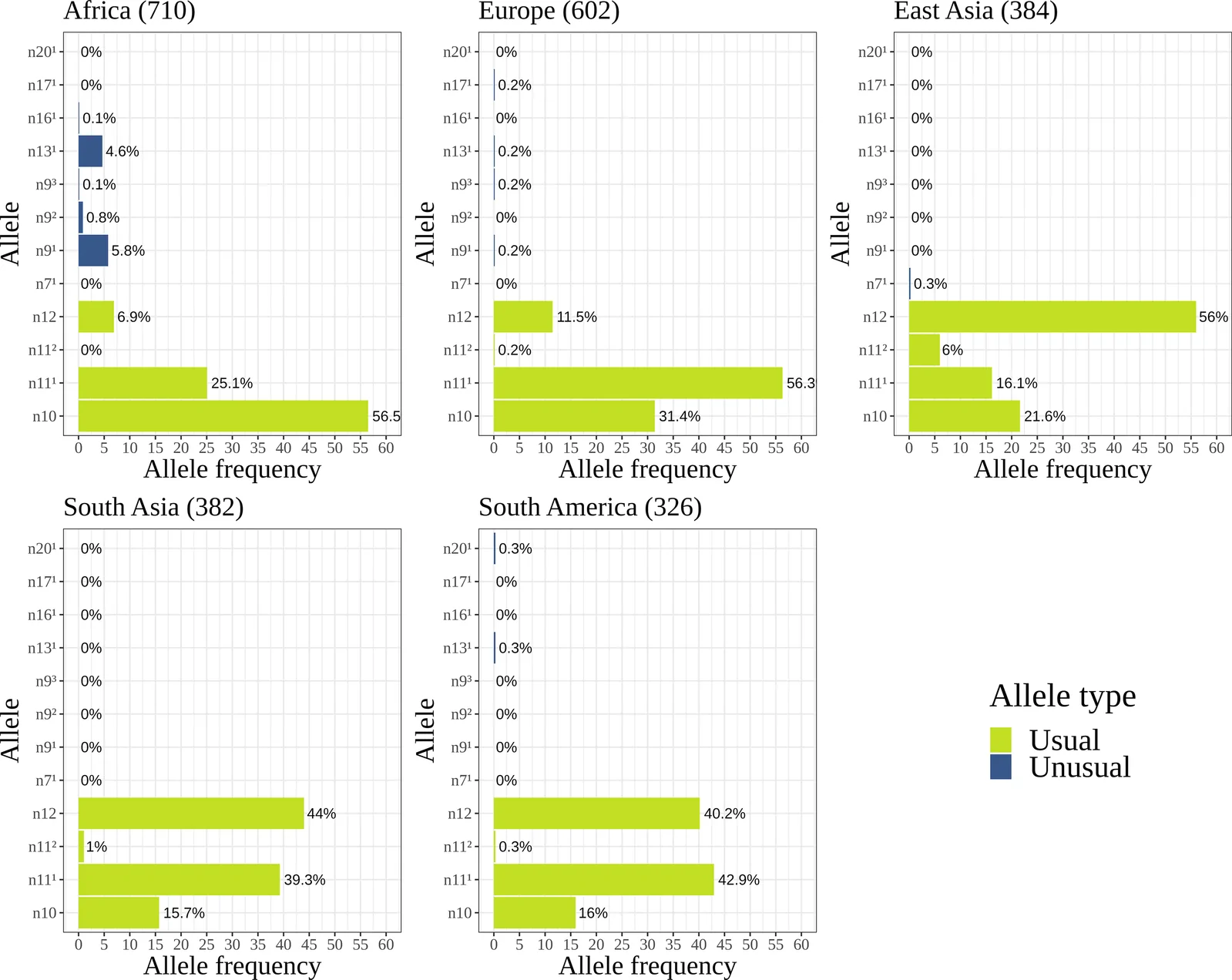
**Barplots of individual frequencies of canon (n10, n111, n112, and n12) and undescribed alleles with unusual number of repeats found across populations.** For each population, the most common allele had a usual number of repeats, ranging from 10 to 12 repeats.

**Figure 14:**
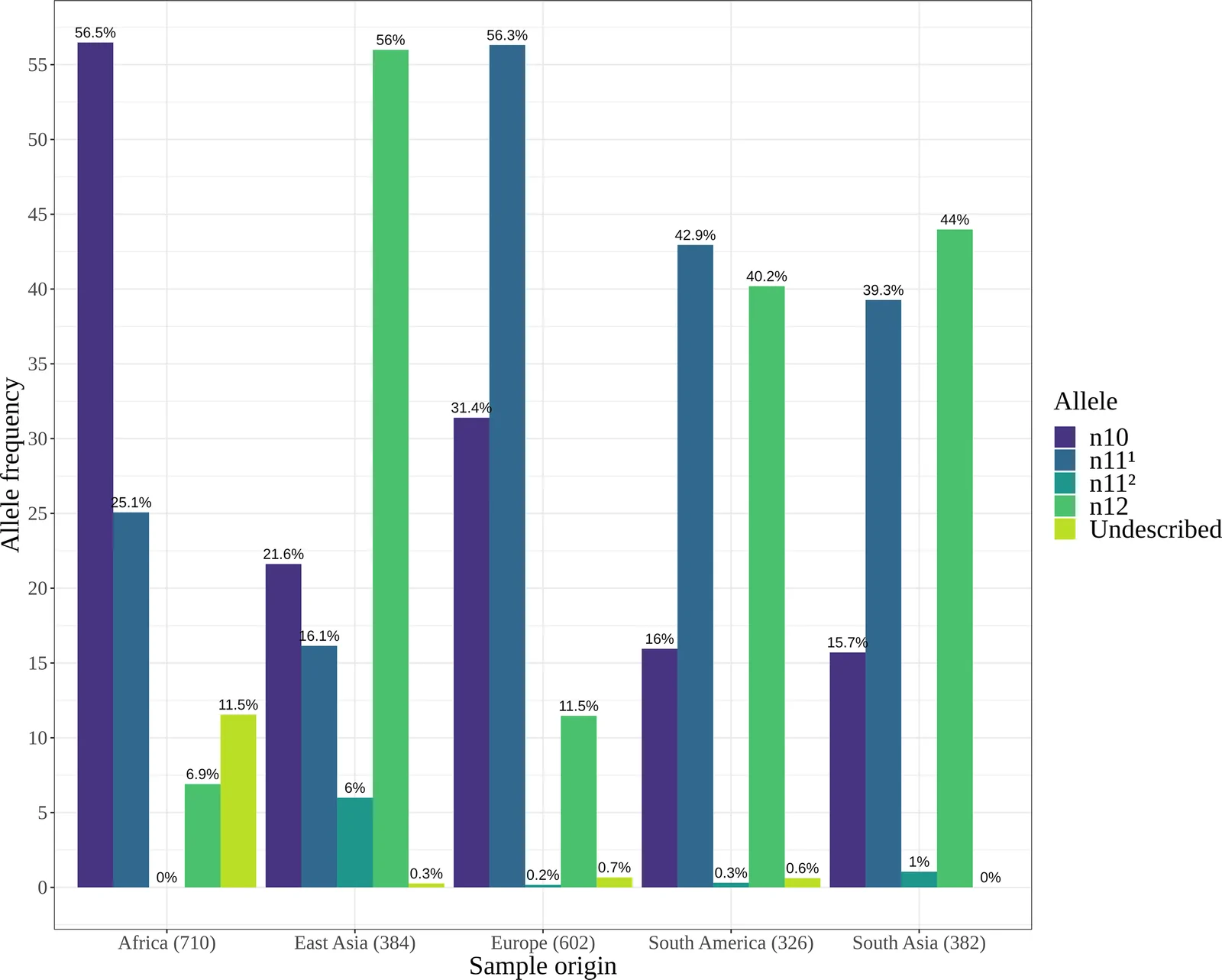
**Barplots of frequencies of canon alleles (n10, n111, n112, and n12) and cumulative frequency of undescribed alleles with unusual number of repeats found.** Alleles with an unusual number of repeats are found mainly in African and African-descent populations.

**Figure 15:**
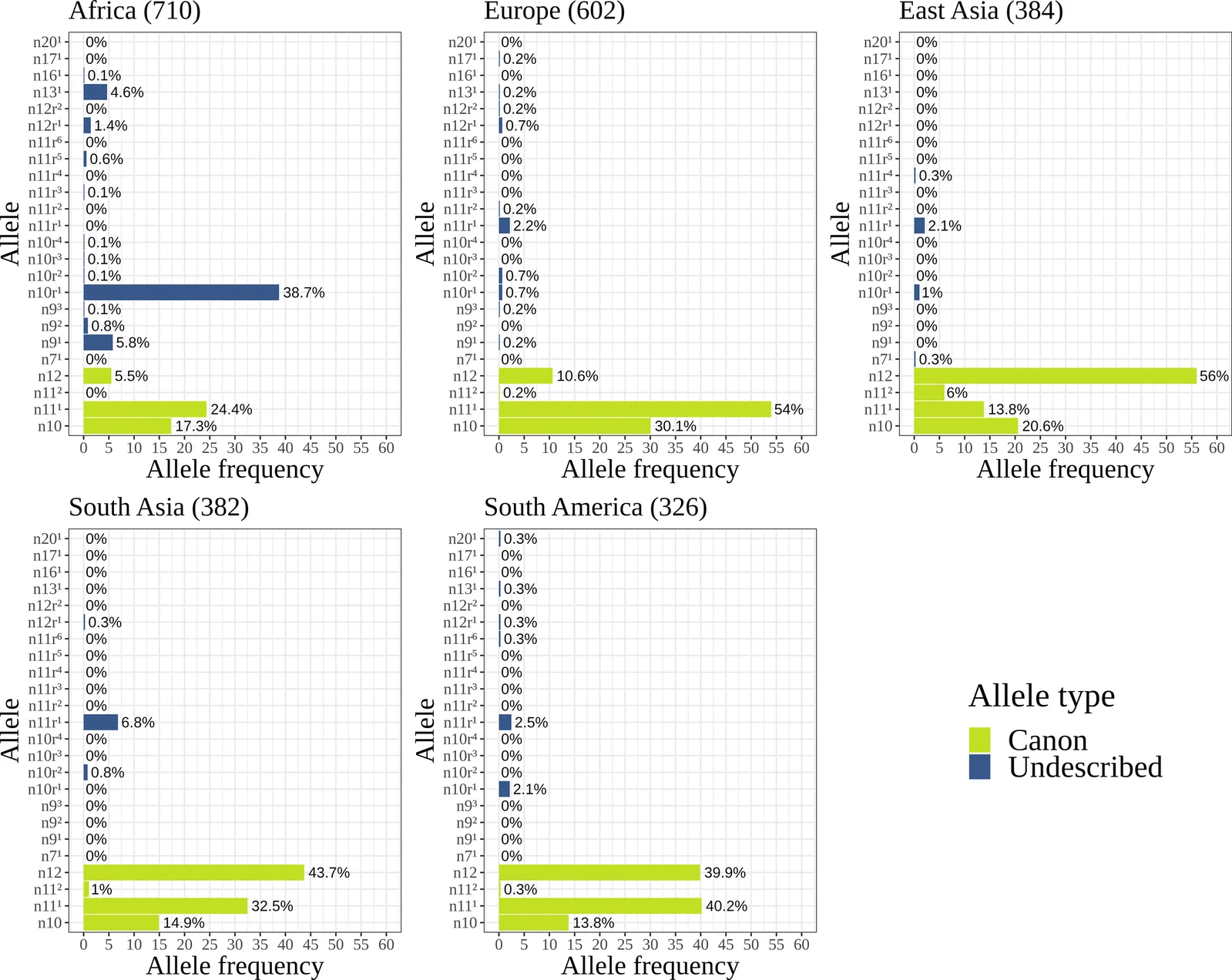
**Barplots of individual frequencies of canon (n10, n111, n112, and n12) and all undescribed alleles found across populations.** Only African and African-descent populations had a change in the most frequent allele compared with Figure 13, with n10r^1^ being more frequent than n10.

**Figure 16:**
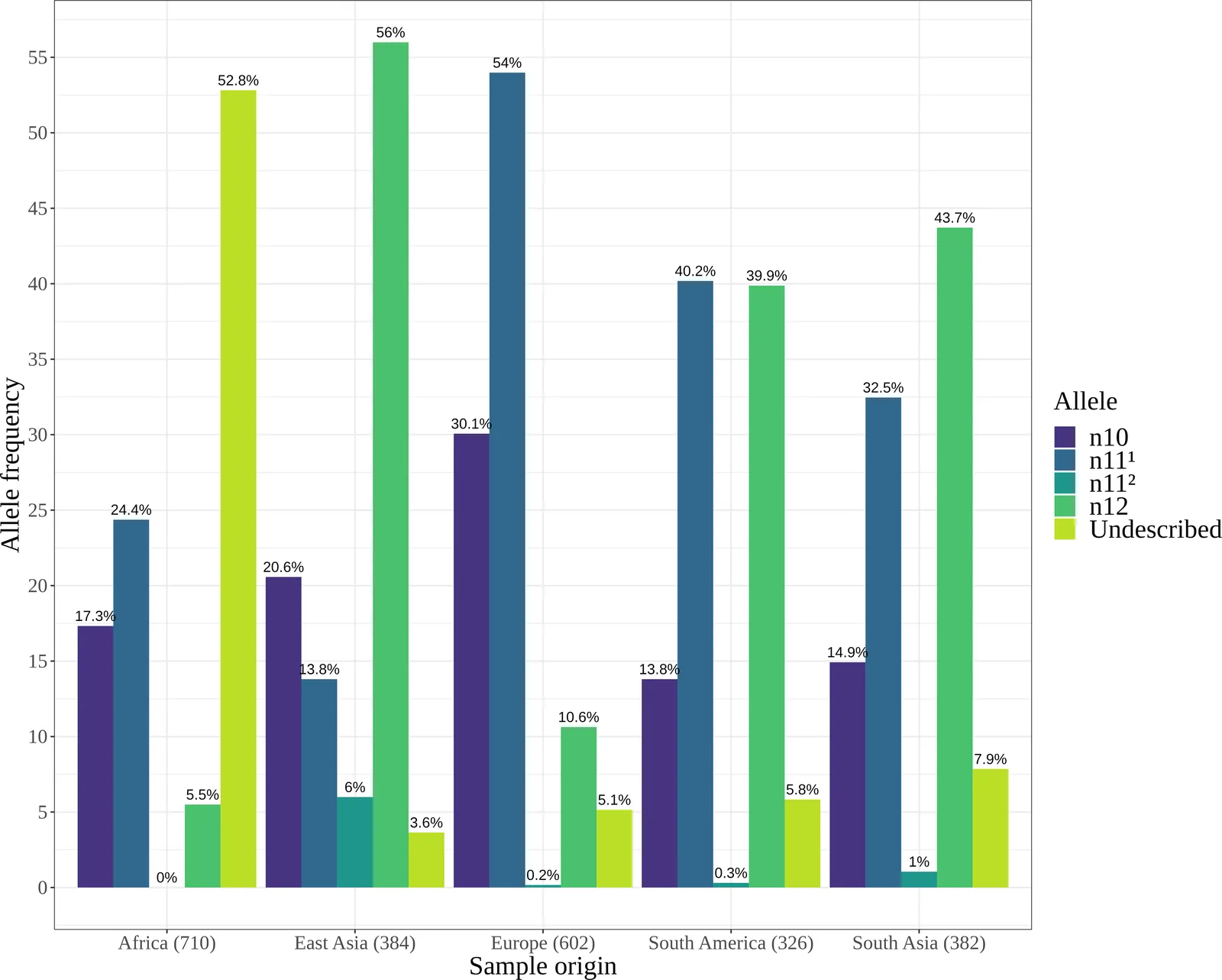
***FLG* allele frequencies.** Shown are bar plots of frequencies of canon (n10, n11^1^, n11^2^, and n12) and previously undescribed alleles found across populations. The majority of undescribed alleles are found in African and African-descent populations.

## Discussion

### Long read sequencing advantages

When compared with other genotyping methods used in a clinical setting, such as TaqMan assays and Sanger Sequencing, which usually only pinpoint the most common *FLG* LOF variants (p.Arg501∗, c2282_2285del, p.Arg2447∗, and p.Ser3247∗) (Khatib et al, 2024), we observed that 28.6% of the LOF variants found using our method were rare variants that are overlooked when using standard genotyping methods. In addition, our method is compatible with DNA extracted from whole blood and buccal swabs, and up to 96 samples can be multiplexed per flow cell. As shown in the recent work of Wong et al (2024), long reads allowed us to accurately phase variants and call compound heterozygous LOF variants in the patient cohort, which is crucial for a comprehensive analysis of AD genetics and the LOF variant contribution to this disease (Lassen et al, 2024; Smith et al, 2006).

During the preparation of this manuscript, Yuda et al (2025) described an approach similar to ours, although with some key differences. Yuda et al (2025) used a single amplicon covering exon 3, whereas we amplified exon 3 in a single amplicon; we also studied 3 additional amplicons: 1 that covers exons 1 and 2, including all introns, and 2 that amplify exon 3 in 2 overlapping amplicons that capture its 5′ and 3′ ends. This ensures that any variants in exon 3 are captured by 2 independent amplicons.

### Pathogenic variants identification and frequency

Our clinical cohort is composed of people living in Belgium, a Western European country with a heterogeneous population and a high number of residents with Southern European or Northern African origins. We identified 8 rare variants in this cohort, with allele frequencies ranging from 0.00060 to 0.0000009; 2 especially rare variants (p.Arg2685∗ and c.3254_3257del) were detected in 1 patient with AD. Their parents originate from Southern Europe, a region for which AD-associated LOF variants have been reported to be less frequent (Khatib et al, 2024). This underlines the importance of a complete analysis of the *FLG* gene to identify risk factors in these populations. Moreover, we identified 1 LOF variant that had not been previously linked to AD or observed in the Genome Aggregation Database (Chen et al, 2024). This particular variant was found on the duplicated repeat 10 found in the 12-repeats *FLG* allele and was only apparent when reads were aligned to our custom genome composed of canonical *FLG* alleles with 10, 11, and 12 repetitions. This may be a very rare variant not sequenced in the Genome Aggregation Database population. Alternatively, it is possible that this variant may be part of a subset of LOF variants missed when sequenced with short reads and mapped to either hg19 or hg38.

Surprisingly, our Belgian cohort included a high proportion of compound heterozygotes. One explanation could be our strict selection of candidates for genetic testing, limited to patients with moderate and severe AD. Recent work investigated the impact of compound heterozygotes in the UK Biobank across phenotypes (Lassen et al, 2024) and recapitulated previous findings that linked *FLG* LOF variants with AD and asthma (Palmer et al, 2006). For AD, these variants increased the risk of developing this disease, with an even greater risk in the case of homozygous or compound heterozygous carriers. However, there was no increase in the risk of developing asthma for heterozygous LOF carriers, but in the case of homozygous/compound heterozygous variants, this risk increased, and they were almost invariably found in conjunction with AD. Although short-read sequencing could be used to determine whether a variant is found in *cis* or in *trans*, this method requires the sequencing of both parents, which, if possible at all, is time consuming and costly. As such, our method of sequencing with long-read technology made it possible for us to unambiguously attribute variants to their haplotype. Therefore, this method may be of particular interest for patient’s follow-up and asthma prevention.

### Identification of undescribed *FLG* alleles

Recently described in the scientific literature (Yuda et al, 2025), undescribed alleles of the *FLG* gene have also been observed in our cohorts, to various degrees. These alleles bore an unusual number of repeats in their sequence (Eaaswarkhanth et al, 2016), ranging from a 7-repeat allele up to a massive 20-repeat allele. Some studies suggest that the number of *FLG* repetitions may have an impact on the severity of AD (Brown et al, 2012), whereas others reported having found no link between them (Fulton et al, 2022). Unfortunately, our study was not designed to address this topical question. A larger number of patients with AD, with a more exhaustive characterization of their condition, would be required to resolve this ambiguity. Nevertheless, our study has shown that our method can effectively identify the different *FLG* alleles. Although there is nothing to suggest a link between these undescribed alleles and AD, because they are found both in patients with AD and healthy controls in our limited cohort (Fisher’s test, *P* = .7013), their existence may increase the difficulty of identifying LOF variants when using short-read sequencing. Indeed, *FLG* LOF variants, in addition to being population specific, are mainly found within a specific allele, such as p.Arg501∗ being found on n11 or c.2282_2285del on n10 (Figure 2). Although we did not find any LOF variants in the undescribed alleles in our limited cohort, given their frequency, it would be parsimonious to assume that there are likely to be examples of LOF variants in these undescribed alleles segregating in the population. It is interesting to note that this and previous work (Thawer-Esmail et al, 2014; Winge et al, 2011) have shown a dearth of *FLG* LOF variants in African and African-descent populations, but the use of long reads has uncovered a wealth of unrecognized diversity in the *FLG* alleles segregating in these populations (Figure 16). Our Rwandan cohort was composed of only 76 samples, with a significant lack of control samples. Given the small sample size, we cannot draw any conclusion regarding the relationship between undescribed *FLG* alleles and the prevalence or severity of AD. Larger cohorts of patients with AD and healthy controls of African origin will be required to determine whether these undescribed haplotypes have any impact on AD.

The most common undescribed *FLG* haplotype we found contained 10 repeats that, after being aligned to the hg19 10-repeat allele, exhibited a high density of single nucleotide variation (∼75 per kb) in a region of around 2 kb. Mapping reads of this haplotype to the 12-repeats *FLG* allele, extracted from the T2T HG002 maternal haplotype, eliminated this single nucleotide variation–rich region and revealed a deletion covering part of repeat 9 and the duplicated repeat 8, indicating that this haplotype is derived from the longer 12-repeat haplotype and has been generated through nonallelic homologous recombination between repeats 9 and 8.1.

As the repetitive region on the third exon of the *FLG* gene has an important impact on the characterization of this region and the genetic diagnosis of AD, being able to accurately identify specific haplotypes is thus of major interest in clinical settings. As such, long-read sequencing is currently the best method to investigate the *FLG* sequence and detect possible clinically relevant variants in all these alleles.

### Improving the characterization of the *FLG* gene

Although long-read sequencing is, for now, the best method to investigate the *FLG* sequence, there are some opportunities to further improve the characterization of this gene. With the release of the T2T assembly (Nurk et al, 2022), a new reference with both maternal and paternal haplotypes with different numbers of *FLG* repetitions, we are able to characterize variants previously hidden in duplications of the 8^th^ and 10^th^ repetitions. Recently, it has been proposed to represent the full variability of the human genome in a human pangenome reference (Liao et al, 2023). Although pangenome graphs are more complex to use for variant calling, in the case of *FLG*, this representation could be of particular interest for clinicians wanting to both characterize the number of CNV in their patient sequences and identify LOF variants specific to each repetition.

However, it must be mentioned that there are also weaknesses in our approach: because it is based on PCR in individuals heterozygous for *FLG* haplotypes that differ markedly in size, there is a possibility of allelic dropout for the larger haplotype due to preferential amplification of the shorter haplotype. This is a more pronounced problem if DNA quality is substandard. We have tried to compensate for this by also amplifying exon 3 in 2 separate PCRs, with primers designed to avoid the most polymorphic region toward the 3′ end of exon 3; nevertheless, there may be recombinant haplotypes where nonallelic homologous breakpoints could potentially impact the amplification with these primers.

## Materials and Methods

### Patients and controls cohort

The patients with AD were selected by geneticists, pediatricians, or dermatologists, and all had mild-to-severe AD, requiring constant care. The DNA was extracted from blood or buccal swabs. The controls came from patient samples collected for other purposes who gave their consent for research activities on leftover DNA. The study was approved by the CHU Liège Ethics committee (reference: 2020/142). The clinical cohort was composed of 117 patients with AD and 75 controls, constituted as follows: 29 patients with AD and 67 controls from people living in Belgium, 20 patients with AD of Irish origin, and 68 patients with AD and 8 controls of Rwandan origin.

### DNA extraction

For Belgian and Rwandan samples, DNA was extracted from peripheral blood using the NucleoMag kit (Macherey-Nagel), according to the manufacturer’s instructions. Alternatively, buccal samples were collected through Isohelix swabs and stored in BuccalFix stabilization buffer. DNA was extracted using the BuccalFix Plus Swab DNA Isolation kit, following the manufacturer's instructions. For Irish samples, DNA was extracted from peripheral blood using the Expand High-Fidelity PCR System (Roche).

### Long range PCR

PCR primers were designed through Primer3 (https://github.com/primer3-org/primer3) and synthesized by Integrated DNA Technologies (http://www.idtdna.com/). Their sequences are described in Table 7.

**Table 7:** Primers Used for PCR Amplification

Primer Name	Forward Primer	Primer Name	Reverse Primer	Description
F1	ACAGAGGGAAGTCACAGAGGGA	R1	GTACGTGAGGAAGCTGGGAAGT	Covers exon 1 and 2
F2	TGGCAAGATCAGCAAGAGGGAT	R2	GCGCAGACTGTCCATGGGT	Covers exon 2 and 5′ end of exon 3
F3	CACACACGGACAGACTGCAC	R3	TCCTGCATGTGATTGTGGGACA	Covers 3′ end of exon 3
F4	TCCCTCTGTGACTTCCCTCTGT	R4	AGAGAAAGATGTGCTAGCCCTGA	Covers whole exon 3

The long-range amplicons encompassed all 3 *FLG* exons as well as the introns. The polymorphic exon 3 was amplified by a single primer pair that spans the complete exon 3; in addition, to guard against allelic dropout, we also designed primer pairs that split exon 3 into 2 parts to reduce amplicon length. This approach also ensures that LOF variants in the coding sequence will be present in 2 independent PCR products. PCR was carried out using the LongAmp Hot Start Taq 2X Master Mix with 200 ng DNA as template, following the manufacturer's instructions. The resultant PCR products were purified through AMPure Beads (Beckman Coulter).

### Long-read sequencing

Long-range PCR products from each individual were mixed at approximately equimolar concentrations. Samples were multiplexed through the Nanopore ligation native barcoding kit (SQK-NBD112.96 or SQK-NBD114.96), following the manufacturer's instructions and sequenced using either 10.4 or 10.4.1 flow cells on a Nanopore GridION in simplex reads. Base calling was done with the “Super accurate model” in Guppy, and barcodes were required at both ends of the molecule for demultiplexing. Libraries used in adaptive sampling were prepared with the Nanopore ligation sequencing kit SQK-LSK114 or the ligation native barcoding kit SQK-NBD114.24, following the manufacturer's instructions.

### Public data cohort

A total of 23 samples with whole-genome sequencing PacBio HiFi FASTQ data were downloaded from the International Genome Sample Resource data portal (Auton et al, 2015), which were sequenced during the 1000 Genomes Project phase 3. Whole-genome sequencing Nanopore sequencing data of 1111 additional samples were downloaded from publicly available datasets (Gustafson et al, 2024; Schloissnig et al, 2025). Samples from the first dataset were prepared using the Nanopore ligation sequencing kit (SQK-LSK110), sequenced using 9.4.1 flow cells on a Nanopore PromethION, and base called using Guppy with the “Super accurate model” with either 5mCG modifications alone or 5mCG and 5hmCG modifications (Gustafson et al, 2024). Data from the second set were prepared using the Nanopore ligation sequencing kit (SQK-LSK110), NEBNext Companion Module for Oxford Nanopore Technologies Ligation Sequencing (E7180S), and in-house AMPure XP beads. Data were then sequenced on 9.4.1 flow cells on a Nanopore PromethION and base called using Guppy with the “Super accuracy model” (Schloissnig et al, 2025). Because these data were already aligned to hg38, we selected reads aligning to the *FLG* region of hg38 to reduce the size of each sample. For quality control, to ensure a correct characterization of each haplotype, samples were discarded if they did not have at least 2 reads covering the whole exon 3. After quality control, 1010 samples were kept for further analysis.

### Ethnicity identification

For our in-house cohort, the ethnicity of samples was classified on the basis of self-report. For our public data cohort, ethnicity was obtained through the samples metadata in the Coriell database. Ethnicity was collected because *FLG* LOF variants have been found to differ in frequency between populations worldwide.

### Data analysis

Our bioinformatics pipeline is described in Figure 17. For our in-house cohort samples, Seqkit (Shen et al, 2016) was used to trim primers, filter out truncated reads, and downsample reads to ∼500-fold coverage. For the public data cohort, aligned reads encoded in BAM format were extracted and transformed by samtools (Danecek et al, 2021) into FASTQ format. For all 1202 samples, Seqkit (Shen et al, 2016) was used for quality control, and reads with a Phred score >10 were selected. These reads were then aligned with minimap2 (Li 2018) to hg19 as well as a custom genome with *FLG* alleles found in hg19 (n10) as well as the T2T HG002 paternal (n11) and maternal (n12) assemblies (Nurk et al, 2022). After that, the aligned data were indexed using samtools (Danecek et al, 2021). Single nucleotide variations were called using Clair3 (Zheng et al, 2022), and LOF variants were annotated with SnpEff (Cingolani et al, 2012). Haplotypes were phased using Whatshap (Martin et al, 2016). Both haplotypes were assembled for each sample with Hifiasm (Cheng et al, 2021) and mapped to hg19 with minimap2 (Li 2018) to confirm the accuracy of the assembly. CNVs were called for each haplotype using Sniffles2 (Smolka et al, 2024) and were used to classify *FLG* alleles. Repetition positions on the third exon were determined using the peptide sequence described in the supplementary materials of the study by Sandilands et al (2007).

**Figure 17:**
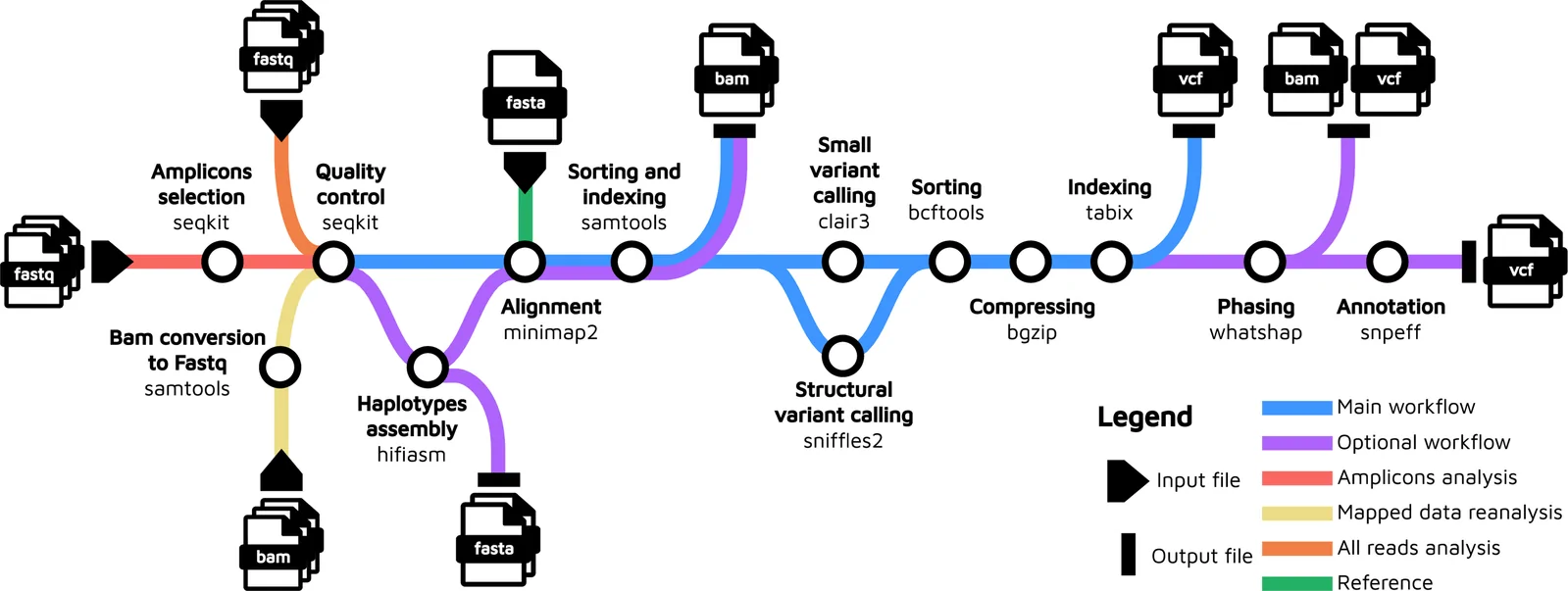
**Bioinformatics pipeline.** Our Nextflow (Di Tommaso et al, 2017) pipeline performs small variant calling (SNPs and indels), structural variant calling (CNVs), phasing, and assembly of haplotypes. Each tool is containerized with Apptainer (Kurtzer et al, 2021). CNV, copy number variation; indel, insertion and deletion.

### Allele identification and characterization

Samples for which an unusual CNV number or position or a high density of SNPs (at least 35 SNPs per repeat) had been called as well as those with a mean depth <10X were inspected manually. This was done by visualizing reads and variants on Integrative Genomics Viewer software (Broad Institute) (Thorvaldsdóttir et al, 2013). The alleles were then labeled either canonical or undescribed, depending on CNV position, length, or SNPs patterns when aligned to our custom *FLG* reference. If labeled as an undescribed allele, the *FLG* repeat sequences were extracted from the assembly of the corresponding haplotype. Then, a BLASTN analysis was performed (Altschul et al, 1990) using repeat sequences from the maternal assembly of T2T HG002 as references (Nurk et al, 2022), because this assembly had the n12 allele. Undescribed alleles were then characterized, depending on the identity score obtained.

### Allele naming convention

To distinguish between alleles with the same number of repeats, we attributed labels following this convention: all allele names start with the letter n, followed by the number of repeats found—for example, n10 for the canon 10-repeat allele. If multiple variations in the order of repeats exist for alleles with the same number of repeats, an index is indicated at the end of the name—for example, n11^1^ for the canon 11-repeat allele with duplicated 8th repeat and n11^2^ for the canon 11-repeat allele with duplicated 10th repeat. This index is incremented by each discovery, meaning that their order depends solely on their order of identification. For alleles with 10, 11, and 12 repeats, if undescribed variations are discovered, to distinguish between the canon—that is, n10, n11^1^, n11^2^, and n12—and undescribed alleles, the letter r is added between the repeat number and the index—for example, n10r^1^ is the first in the list of undescribed 10-repeat alleles that we identified.

### Validation of c.10472del with an alternative sequencing technology

The region encompassing the c.10472del was amplified through an ∼800 bp amplicon (forward GGGATCCATGTCTCTCTCCTGC, reverse CACAGGGAAAAGCTGGGCC) using DNA from the carrier as well as the genome in a bottle sample HG002. The PCR product from the carrier was initially sequenced on an ABI 3730. The products were also cloned into a TOPO XL-2 (Thermo Fisher Scientific) plasmid, followed by sequencing with Nanopore using an R10.4.1 MinION flow cell and super accurate base calling through Dorado, version 1.1.1. The plasmids were also sequenced on the Illumina short-read platform through Nextera XT (Illumina) library prep and sequencing on a NovaSeq 6000 with a flow cell SP300, paired-end 150 (2 × 150 bp reads). The resultant reads were mapped to the maternal haplotype of the HG002 assembly (GCA_018852615.1_HG002.pri.mat.f1_v2), which also carries the N12 FLG allele.

## Ethics Statement

The analyses were performed as part of a development and validation study, approved by the CHU Liège Ethics Committee (reference: 2020/142), and as routine testing at the request of the treating physicians.

## Data Availability Statement

Raw reads from our in-house cohort have been submitted to the European Nucleotide Archive; accession https://www.ebi.ac.uk/ena/browser/view/ERP180693. Public data can be accessed through these links https://www.internationalgenome.org/data-portal/sample, https://ftp.1000genomes.ebi.ac.uk/vol1/ftp/data_collections/1KG_ONT_VIENNA/. and https://s3.amazonaws.com/1000g-ont/index.html. The code used to process the data can be found at https://gitlab.uliege.be/Victor.Grentzinger/longseq.

## ORCIDs

Victor Grentzinger: http://orcid.org/0009-0006-7868-5953

Maria Artesi: http://orcid.org/0009-0002-5003-6143

Leonor Palmeira: http://orcid.org/0009-0004-8750-6692

Laura Helou: http://orcid.org/0000-0003-0608-5861

Benoit Charloteaux: http://orcid.org/0000-0003-4282-9688

W.H. Irwin McLean: http://orcid.org/0000-0001-5539-5757

Aileen Sandilands: http://orcid.org/0009-0000-9910-6304

Maeve A. McAleer: http://orcid.org/0000-0001-9958-6504

Alan D. Irvine: http://orcid.org/0000-0002-9048-2044

Annette Uwineza: http://orcid.org/0000-0003-1749-0395

Alice Amani Uwajeni: http://orcid.org/0009-0004-3518-5954

Keith Durkin: http://orcid.org/0000-0001-7475-6618

Vincent Bours: http://orcid.org/0000-0002-5934-6247

## Conflict of Interest

SO is chief executive officer of Theratopix Pharma. VB and SO are both members of the board and stock owners of Theratopix Pharma. The remaining authors state no conflict of interest.
